# Identification of a Novel Link between the Protein Kinase NDR1 and TGFβ Signaling in Epithelial Cells

**DOI:** 10.1371/journal.pone.0067178

**Published:** 2013-06-26

**Authors:** Isabelle Pot, Shachi Patel, Lili Deng, Amrita Singh Chandhoke, Chi Zhang, Azad Bonni, Shirin Bonni

**Affiliations:** 1 Southern Alberta Cancer Research Institute and Department of Biochemistry and Molecular Biology, University of Calgary, Calgary, Alberta, Canada; 2 Department of Neurobiology, Harvard Medical School, Boston, Massachusetts, United States of America; 3 Department of Anatomy and Neurobiology, Washington University School of Medicine, St Louis, Missouri, United States of America; IISER-TVM, India

## Abstract

Transforming growth factor-beta (TGFβ) is a secreted polypeptide that plays essential roles in cellular development and homeostasis. Although mechanisms of TGFβ-induced responses have been characterized, our understanding of TGFβ signaling remains incomplete. Here, we uncover a novel function for the protein kinase NDR1 (nuclear Dbf2-related 1) in TGFβ responses. Using an immunopurification approach, we find that NDR1 associates with SnoN, a key component of TGFβ signaling. Knockdown of NDR1 by RNA interference promotes the ability of TGFβ to induce transcription and cell cycle arrest in NMuMG mammary epithelial cells. Conversely, expression of NDR1 represses TGFβ-induced transcription and inhibits the ability of TGFβ to induce cell cycle arrest in NMuMG cells. Mechanistically, we find that NDR1 acts in a kinase-dependent manner to suppress the ability of TGFβ to induce the phosphorylation and consequent nuclear accumulation of Smad2, which is critical for TGFβ-induced transcription and responses. Strikingly, we also find that TGFβ reciprocally regulates NDR1, whereby TGFβ triggers the degradation of NDR1 protein. Collectively, our findings define a novel and intimate link between the protein kinase NDR1 and TGFβ signaling. NDR1 suppresses TGFβ-induced transcription and cell cycle arrest, and counteracting NDR1's negative regulation, TGFβ signaling induces the downregulation of NDR1 protein. These findings advance our understanding of TGFβ signaling, with important implications in development and tumorigenesis.

## Introduction

The transforming growth factor beta (TGFβ) family of cytokines regulates a wide array of biological responses that are critical for proper development and homeostasis [1], [2], [3], [4]. Deregulation of TGFβ-mediated responses contributes to the pathogenesis of diverse disease processes from pulmonary and renal fibrosis to cancer [5], [6], [7], [8], [9]. A widely studied and key biological effect of TGFβ is the inhibition of hematopoietic and epithelial cell proliferation [10], [11], [12], [13], which has important consequences in cancer biology. Several types of carcinomas acquire resistance to TGFβ-induced cell cycle arrest, leading to uncontrolled cell proliferation [10], [11], [12], [13], [14].

TGFβ ligands form heteromeric complexes with type I and II transmembrane TGFβ receptors, which have intrinsic serine/threonine kinase activities [15], [16], [17], [18], [19]. The type II kinase transphosphorylates the type I receptor in a glycine-serine rich motif, thereby stimulating the type I kinase activity [20], [21], [22]. The Smad family of intracellular signaling proteins is critical for transducing TGFβ signals from the cell surface to the nucleus to regulate gene expression and consequent cellular processes [7], [23], [24]. In particular, the TGFβ-stimulated type I receptors associate and phosphorylate the receptor-regulated Smad (R-Smad) proteins Smad2 and Smad3 on the C-terminal two serine residues in the SSXS motif [23], [24], [25], [26]. The phosphorylated R-Smads then form a heteromeric complex with the common partner Smad4, and the R-Smad/Smad4 complex accumulates in the nucleus and binds to specific binding elements within promoters of TGFβ responsive genes [26], [27], [28]. The R-Smad/Smad4 complex acts together with other proteins to induce or repress transcription of responsive genes [29], [30], [31].

The transcriptional protein SnoN has emerged as a key regulator of TGFβ signaling and responses [32], [33], [34], [35]. SnoN associates with R-Smad2/3 and Smad4 and thereby regulates TGFβ-induced transcription [36], [37], [38]. SnoN activates or represses TGFβ-induced transcription, leading to divergent biological responses in a cell type- or context-dependent manner [33], [34], [39], [40]. The critical role of SnoN in TGFβ signaling suggests that identifying novel SnoN-associating proteins should enhance our understanding of TGFβ responses.

NDR1 is a member of the evolutionary conserved NDR (nuclear Dbf2-related) family of serine-threonine kinases that form a subgroup of AGC kinases [41]. NDR1 and the closely related family member NDR2 regulate critical cellular processes including cell proliferation, apoptosis and differentiation [42], [43], [44], [45], [46]. The expression of NDR kinases is deregulated in carcinomas including breast, lung and prostate cancer [47], [48]. Interestingly, NDR kinases have been proposed to harbor positive or negative roles in tumorigenesis [47], [48]. Whether these kinases regulate specific signaling pathways has remained largely unexplored [48].

Here, we identify NDR1 as a novel SnoN-interacting protein. We find that NDR1 inhibits TGFβ-induced transcription and cell cycle arrest. NDR1 inhibits Smad2 phosphorylation, providing the basis for NDR1 regulation of TGFβ responses. Remarkably, TGFβ reciprocally promotes the degradation of NDR1, thereby providing a counterbalance to NDR1-inhibition of TGFβ signaling. Collectively, our findings point to a novel and intimate link between the protein kinase NDR1 and TGFβ signaling, with profound effects on the regulation of gene expression and cell proliferation.

## Results

### NDR1 Associates with the TGFβ Signaling Protein SnoN

**Figure 1:**
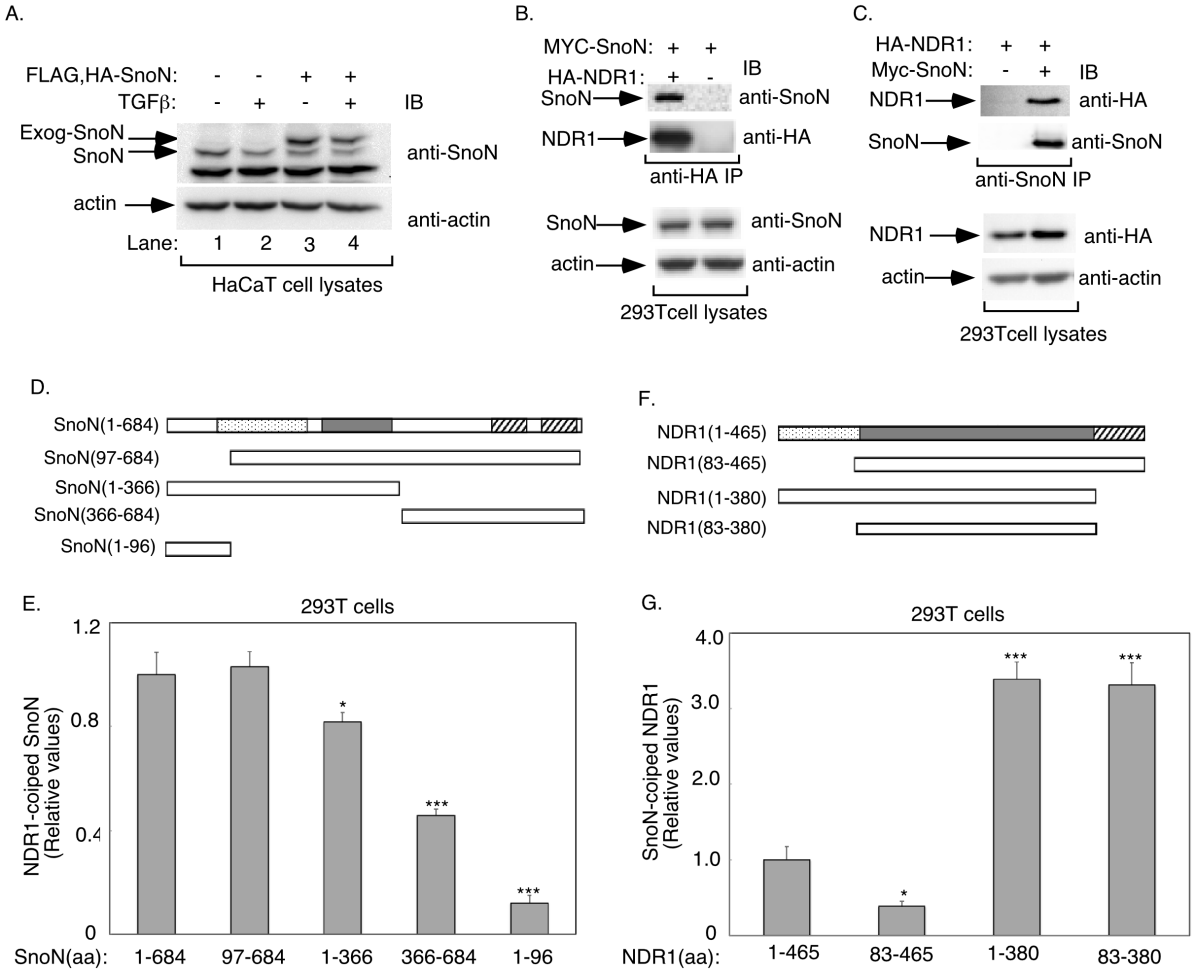
NDR1 is a novel SnoN-interacting protein. **A.** Lysates of untreated or TGFβ-treated HaCaT cells expressing FLAG, HA-SnoN or control vector were immunoblotted with the SnoN or actin antibody. TGFβ similarly reduced the abundance of endogenous and exogenous SnoN in HaCaT cells. **B.** Lysates of 293T expressing MYC-SnoN alone or together with HA-NDR1 were subjected to immunoprecipitation with the HA antibody followed by immunoblotting with the SnoN or HA antibody. Total lysates were also subjected to immunoblotting with the SnoN or actin antibody, the latter to serve as a loading control. **C.** Lysates of 293T cells expressing HA-NDR1 alone or together with MYC-SnoN were subjected to immunoprecipitation with the SnoN antibody followed by immunoblotting with the HA or SnoN antibody. Lysates were also immunoblotted with the HA or actin antibody. NDR1 formed a complex with SnoN. **D.** A schematic diagram showing the wild type (amino acid (aa) 1–684) and four deletion mutants of SnoN. The dotted area represents the ski/sno/dac (DACH) domain, the shaded area the SAND domain, and the striped areas the helical dimerization domains [33]. **E.** Lysates of 293T cells expressing Rluc in fusion with wild type or a series of SnoN mutants, as shown in D, alone or together with HA-NDR1 were subjected to immunoprecipitation with the HA antibody followed by luciferase assays to determine the levels of Rluc-SnoN fusion proteins in the NDR1 immunoprecipitates. Aliquots of cell lysates were also assayed for luciferase activity as a measure of Rluc-SnoN expression. The expression of HA-NDR1 in aliquots of immunoprecipitates (10%) and cell lysates was confirmed by immunoblotting using the HA antibody (data not shown). NDR1-associated Rluc-SnoN luciferase activity was normalized to Rluc-SnoN and NDR1 expression. Data are presented as the mean+SEM (n = 4) of NDR1-associated Rluc activity relative to Rluc activity associated with NDR1 in the case of the wild type Rluc-SnoN fusion protein. **F.** A schematic diagram showing the wild type (aa1–465) and three deletion mutants of NDR1. The dotted area represents the N-terminal regulatory domain, the shaded area the kinase domain, and the striped area the C-terminal regulatory domain. **G.** Lysates of 293T cells expressing Rluc in fusion with wild type or a series of NDR1 mutants, as in F, alone or together with MYC-SnoN, were subjected to immunoprecipitation using the MYC or SnoN antibody. Immunoprecipitates and cell lysates were subjected to luciferase assays, SnoN immunoblotting (Data not shown), and data analyses as described in E. Data are presented as the mean+SEM (n = 7) of SnoN-associated Rluc activity expressed relative to SnoN-associated Rluc activity in the case of wild type NDR1-Rluc. *, or *** indicates significant difference as compared to wild type SnoN-Rluc (E) or NDR1-Rluc (G) at p<0.05 or p<0.001, respectively (ANOVA).

To gain new insights into the signaling mechanisms that control TGFβ responses, we focused on identifying proteins that interact with SnoN, a key component in TGFβ signaling. We used a tandem affinity purification (TAP) approach to immunopurify SnoN in human HaCaT keratinocytes, in which we stably expressed the double epitope-tagged version of SnoN (FLAG, HA-SnoN). To identify true SnoN associated proteins, we used cells expressing epitope-tagged SnoN at levels equivalent to those of endogenous SnoN (Figure 1A). Interestingly, stable expression of SnoN reduced the level of endogenous SnoN in these cells, further normalizing the level of SnoN between SnoN-expressing and control vector-transfected cells (Figure 1A, compare endogenous SnoN in lane 1 and exogenous SnoN in lane 3). Exposure of HaCaT cells to TGFβ led to the downregulation of endogenous as well as stably expressed SnoN, suggesting that TGFβ signaling behaves normally in HaCaT cells expressing epitope-tagged SnoN [37], [38], [49], [50]. We performed tandem affinity purification (TAP) by sequential FLAG and HA immunoprecipitation of lysates of epitope-tagged SnoN-expressing HaCaT cells and control HaCaT cells followed by mass spectrometry of immunocomplexes [51], [52]. SnoN and known SnoN-interacting proteins including Ski and Smad4 were immunopurified from SnoN-expressing cells, confirming the validity of the purification procedure (Table 1). We also identified novel SnoN-interacting proteins (Table 1). Among these proteins, we focused on the protein kinase NDR1 (also known as STK38).

**Table 1:** Mass spectrometry data of SnoN-interacting proteins in HaCaT cells.

Identified proteina,b	gi number	Mass	Score^c^	Number of peptides matched	Ion score range
*Gel slice 1*					
SnoN	4885599	76955	137	10	7–69
RBM10	12644371	103396	80	4	6–63
Ski	4506967	79955	64	5	21–49
*Gel slice 2*					
SnoN	4885599	76955	214	19	8–68
Skb1Hs/PRMT5^d^	232410	72740	161	12	2–82
STK38/NDR1	6005814	54155	115	9	17–52
PTP1B^d^	4505995	52609	106	8	4–53
Smad4	4885457	60401	104	12	5–62
α-tubulin	340021	50120	87	3	17–68
*Gel Slice 3*					
No significant hits					

a Identified proteins represent peptides only present in the SnoN-expressing samples, and not in the control cells.

b Trypsin, immunoglobulin and keratin are considered contaminants and are omitted from this list.

c only identified proteins with a score of ≥50 are shown here.

d These identified proteins were also present in the control cells, but the number of peptides matched and overall score was much lower in the control cells than in the SnoN-expressing samples.

NDR1 and its close relative NDR2 regulate several biological processes including cell proliferation, apoptosis and differentiation [42], [43], [44], [45], [46]. However, whether these kinases regulate specific growth factor signaling pathways has remained incompletely understood. Therefore, we further characterized the interaction of NDR1 with the TGFβ signaling protein SnoN. In co-immunoprecipitation assays, we confirmed that SnoN and NDR1 formed a complex (Figure 1B, C). We next used the fusion of NDR1 or SnoN with the *Renilla* luciferase (Rluc) protein to assess the interaction of endogenous SnoN or endogenous NDR1, respectively, using *Renilla* luciferase activity as the readout [53]. We found that endogenous NDR1 robustly interacted with Rluc-SnoN (Figure S1A). Likewise, endogenous SnoN strongly interacted with Rluc-NDR1 (Figure S1B). Consistent with these results, endogenous NDR1 formed a complex with endogenous SnoN in the absence or presence of TGFβ in 293T cells (Figure S1C). Together, these data suggest that NDR1 associates with SnoN in epithelial cells.

To gain further insight and evidence for the specificity of the SnoN-NDR1 association, we mapped the structural determinants of SnoN that are required for its interaction with NDR1 (Figure 1D). We used a series of Rluc-SnoN mutants in coimmunoprecipitation assays to enable quantitative assessment of the effect of mutations of SnoN on its interaction with expressed NDR1 in 293T cells. Removal of the N-terminal 96 amino acids did not affect SnoN's association with NDR1 (Figure 1E). Consistent with these results, the N-terminal 96 amino acids of SnoN failed to associate with NDR1. Interestingly, we found that deletion of amino acid residues 366–684 or 1–366 decreased significantly the ability of SnoN to interact with NDR1 (Figure 1E).

We also identified the regions in NDR1 that specify its association with SnoN (Figure 1F). We used the Rluc-NDR1 fusion and its mutants in these experiments. Deletion of the N-terminal 82 amino acid residues reduced the ability of NDR1 to associate with SnoN (Figure 1G). Interestingly, deletion of the C-terminal regulatory region alone or together with the N-terminal domain dramatically increased the ability of NDR1 to coimmunoprecipitate with SnoN (Figure 1G). Together, these data suggest that a region within the kinase domain specifies the association of NDR1 with SnoN, and the C-terminal regulatory region may interfere with the NDR1-SnoN interaction.

### NDR1 Regulates TGFβ-dependent Transcription

**Figure 2:**
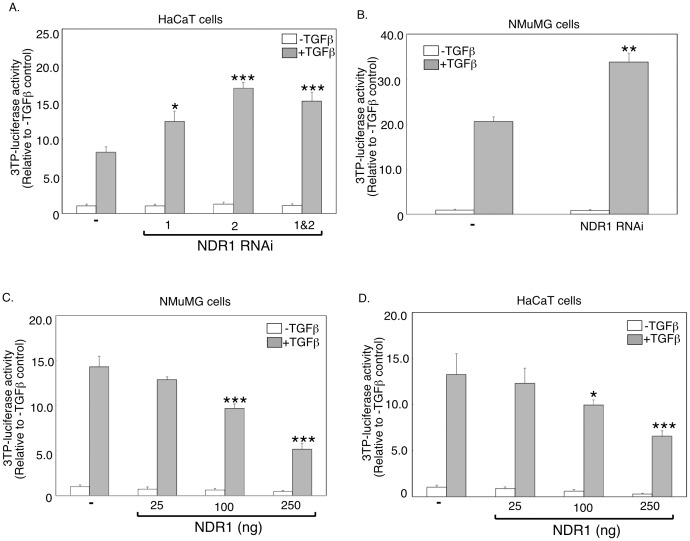
NDR1 inhibits TGFβ-induced transcription. **A.** Lysates of untreated or TGFβ-treated HaCaT cells transfected with the TGFβ-responsive 3TP-*Firefly* luciferase reporter and CMV-*Renilla*-luciferase reporter, as a transfection efficiency control, together with the control RNAi vector (−), or NDR1 RNAi NDR1i-1, NDR1i-2 plasmid alone or together, were subjected to dual luciferase assays. Data are presented as the mean+SEM (n = 4) of normalized-3TP-luciferase activity expressed relative to that of the untreated control. **B.** Lysates of NMuMG cells transfected with reporters as in A together with the control RNAi plasmid or the NDR1i-2 plasmid, and analyzed as in A. Data are presented as the mean+SEM (n = 3) of luciferase activity expressed relative to the untreated control. **C.** Lysates of untreated or TGFβ-treated NMuMG cells transfected with reporters as in A, together with a control vector (−) or increasing concentrations of an NDR1 expression plasmid, were subjected to dual luciferase assays and data analysis as in A. **D.** Lysates of untreated or TGFβ-treated HaCaT cells transfected as described for NMuMG cells in C except for using the CMV-β-galactosidase expression plasmid as a transfection efficiency reporter, were subjected to luciferase and β-galactosidase assays. For each experiment, luciferase activity was normalized as in A. Data in C and D are presented as the mean+SEM (n = 5) of 3TP-luciferase activity expressed relative to the untreated control. *, **, or *** indicates significant difference from the TGFβ-treated control at p<0.05, p<0.01, or p<0.001, respectively (ANOVA).

The finding that NDR1 associates with SnoN raised the important question of whether NDR1 regulates TGFβ signaling. The plasminogen activator inhibitor 1 (PAI-1) is a TGFβ-responsive immediate early gene that has been linked to the control of cell proliferation [40], [54], [55], [56]. We characterized the role of NDR1 in TGFβ-induced transcription employing the widely used 3TP-luciferase reporter gene containing TGFβ-responsive promoter elements of the PAI-1 gene [56]. We first determined the effect of inhibition of endogenous NDR1 on TGFβ-induced transcription. We used RNA interference (RNAi) to induce knockdown of endogenous NDR1 in epithelial cells. Two short hairpin RNAs (shRNAs) targeting distinct regions of NDR1 mRNA induced efficient knockdown of NDR1 protein in 293T cells (Figure S2A). Importantly, in reporter assays, knockdown of NDR1 using the two shRNAs singly or in combination significantly enhanced the ability of TGFβ to induce expression of the 3TP-luciferase reporter gene in HaCaT keratinocytes (Figure 2A). Knockdown of NDR1 in NMuMG mammary epithelial cells with expression of NDR1 shRNAs also increased TGFβ-induced 3TP-luciferase-reporter gene expression (Figure 2B). In complementary reporter assays, expression of NDR1 reduced in a dose-dependent manner the ability of TGFβ to induce expression of the 3TP-luciferase gene in NMuMG cells (Figure S2B and Figure 2C). Similarly, NDR1 repressed the ability of TGFβ to induce the expression of the 3TP-luciferase reporter gene in HaCaT cells (Figure 2D). Thus, based on knockdown and gain of function analyses, we conclude that NDR1 inhibits the ability of TGFβ to induce transcription.

**Figure 3:**
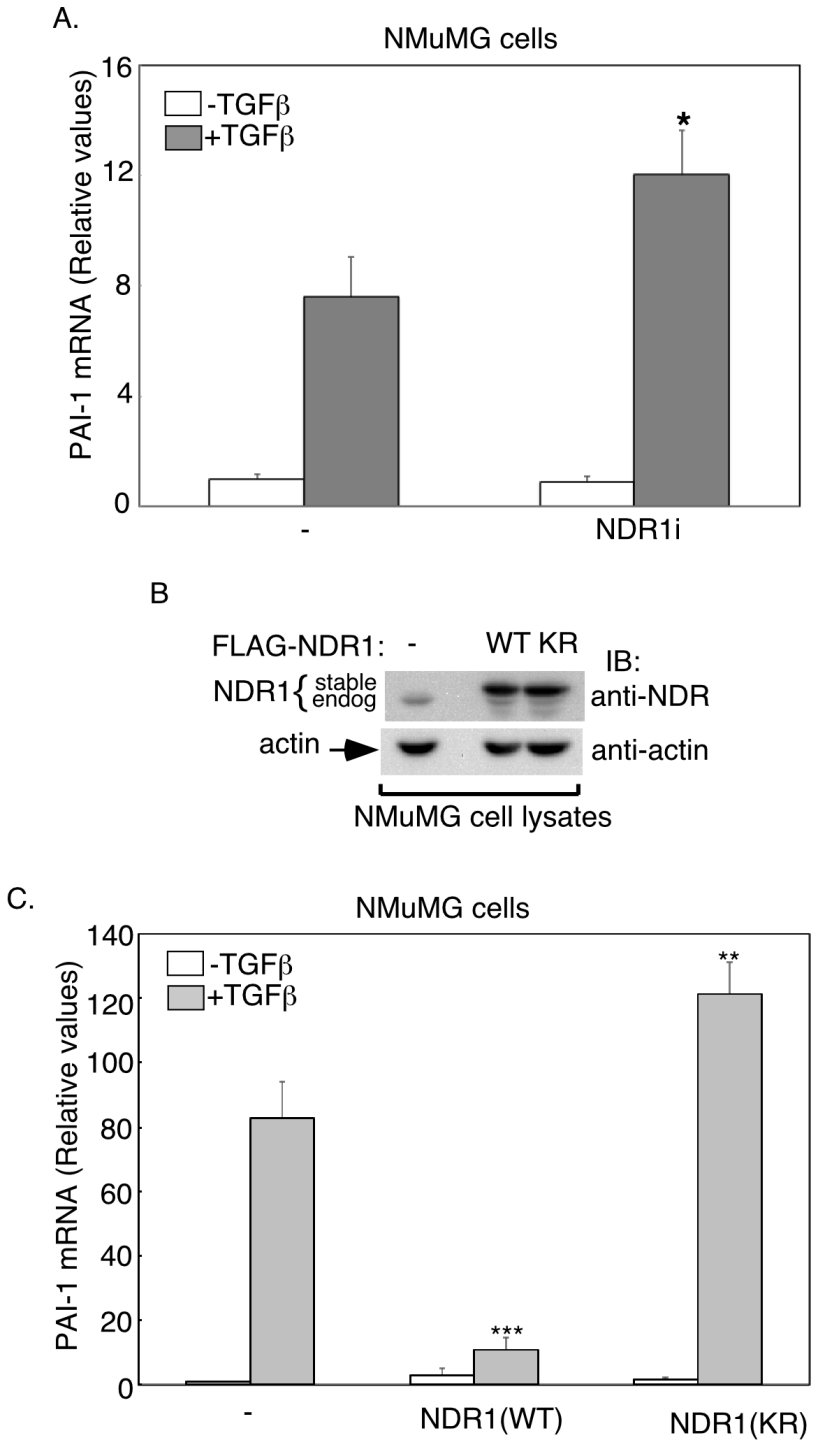
NDR1 represses TGFβ-induction of endogenous PAI-1 gene expression. **A.** RNA extracts of untreated or TGFβ-treated NMuMG cells transfected with a control RNAi plasmid or the combination of NDR1i-1 and NDR1i-2 RNAi plasmids were subjected to quantitative RT-PCR to determine the abundance of PAI-1 mRNA, where GAPDH mRNA was used as an internal control. Data are presented as the mean+SEM (n = 5) of GAPDH-normalized PAI-1 mRNA abundance relative to untreated control. **B.** Lysates of NMuMG cells expressing FLAG-tagged wild type NDR1 (WT) or kinase-inactive NDR1 in which Lysine 118 is mutated to arginine (KR), or vector control were subjected to immunoblotting using the NDR1 or actin antibody, with the latter serving as a loading control. **C.** RNA extracts from untreated or TGFβ-treated NMuMG cells expressing wild type or kinase-inactive NDR1 or the vector control were subjected to quantitative RT-PCR analysis of PAI-1 and GAPDH mRNA as described in A. Data are presented as the mean+SEM (n = 3) of relative GAPDH-normalized PAI-1 mRNA abundance as in A. *, **, or *** indicates significant difference from the TGFβ-treated control at p<0.05, p<0.01, or p<0.001, respectively (ANOVA).

We next characterized the role of NDR1 in the regulation of TGFβ-induced expression of the endogenous PAI-1 gene. As expected, TGFβ stimulation of control-transfected NMuMG cells increased the abundance of endogenous PAI-1 mRNA as assessed by quantitative real time PCR. Knockdown of endogenous NDR1 in NMuMG cells significantly enhanced the ability of TGFβ to increase the abundance of PAI-1 mRNA (Figure 3A). We also measured the ability of TGFβ to induce PAI-1 expression in NMuMG cells stably expressing wild type NDR1 (WT) or a kinase-inactive version of NDR1 in which Lysine 118 was mutated to arginine (KR) (Fig. 3B). The expression of wild type NDR1 blocked TGFβ-induced PAI-1 mRNA expression in NMuMG cells (Figure 3C). In contrast, the kinase-inactive NDR1 enhanced the ability of TGFβ to increase the abundance of PAI-1 mRNA (Figure 3C). These data suggest that the kinase-inactive NDR1 enhanced TGFβ-induced PAI-1 gene expression by acting in a dominant negative fashion to block the ability of endogenous NDR1 to antagonize TGFβ-induced transcription. Together, these data suggest that NDR1 acts in a kinase-dependent manner to negatively regulate TGFβ-induced transcription.

### NDR1 Antagonizes TGFβ-induced Cell Cycle Arrest

**Figure 4:**
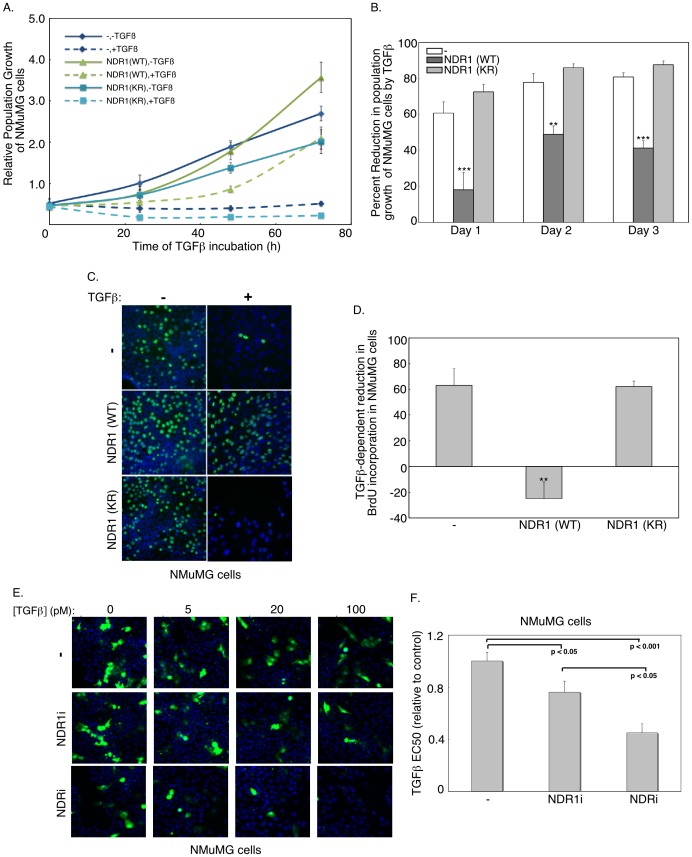
NDR1 suppresses TGFβ-repression of cell proliferation. **A.** Population growth curves of NMuMG cells expressing wild type (WT) or kinase-inactive (KR) NDR1 or the vector control (−) analyzed using phase-contrast microscopy after one, two or three days of culturing in the absence (solid lines) or presence (broken lines) of TGFβ. Each point is the mean±SEM (n = 7) of cell population normalized to cell population seeding. **B.** The effect of TGFβ on cell population growth curves for NMuMG expressing NDR1 or the vector control as in A was analyzed as the difference of untreated and TGFβ-treated cell populations as a percent of untreated cell counts. Each point is the mean+SEM (n = 7) of the percent decrease in population growth by TGFβ. **C.** Representative fluorescence microscopy images of untreated or 48h-TGFβ-treated NMuMG cells expressing wild type or kinase-inactive NDR1 or the vector control that were incubated for 1 h with bromodeoxyuridine (BrdU) and subjected to indirect immunofluorescence using the BrdU antibody and a Cy2-conjugated secondary antibody (green), and labeling with the DNA Hoechst dye (blue) **D.** For each cell type as in C, percent reduction of BrdU-labeled cells in response to TGFβ was quantified as in B. Data are presented as the mean+SEM (n = 3) of the percent reduction in BrdU incorporation in cells in response to TGFβ. **E.** Representative fluorescence images of GFP-expressing (green) and DNA dye (Hoechst) (blue)-labeled NMuMG cells transfected with control RNAi plasmid or the combination of NDR1i-1 and NDR1i-2 (NDR1i) RNAi plasmids alone or together with the combination of NDR2i-1 and NDR2i-2 RNAi (NDRi) plasmids, and incubated one day post transfection with 0, 5, 20 and 100 pM TGFβ for 36 h. **F.** A target activation algorithm accompanying the Cellomics KSR instrument used to capture images including those shown in E was used to determine population growth of GFP-positive cells in 96 well plates. For each experiment, triplicate average population growth of GFP-positive cells was plotted versus TGFβ concentration and fitted using log transformation to obtain the effective concentration of TGFβ leading to 50% reduction of population growth of GFP-expressing cells (EC50). Data are presented as the mean+SEM of EC50 values expressed relative to the NMuMG cells transfected with the vector control from six (- and NDRi) or five (NDR1i) independent experiments. The width of each fluorescence micrograph in C and E corresponds to 330 µM. **, or *** indicates significant difference from the control at p<0.01, or p<0.001, respectively (ANOVA).

The finding that NDR1 inhibits TGFβ-induced gene expression led us to ask whether NDR1 might also impact biological processes regulated by TGFβ. NMuMG cells undergo growth arrest in response to TGFβ stimulation [57]. We analyzed the growth rate curves of NMuMG cells stably expressing wild type NDR1 or kinase-inactive NDR1 protein (KR). Cells were plated and left untreated or incubated with TGFβ and counted after one, two or three days (Figure 4A, B). As expected, TGFβ reduced the population growth of control-transfected cells (Figure 4A, B). However, expression of wild type but not the kinase-inactive NDR1 significantly inhibited the ability of TGFβ to suppress the population growth of NMuMG cells (Figure 4A, B). These data suggest that NDR1 impairs TGFβ-induced cell cycle arrest in a kinase-dependent manner. NDR1 similarly opposed TGFβ suppression of population growth of NMuMG cells when we employed automated cell counts using the Cellomics KSR instrument whereby NMuMG cells were labeled with the DNA dye bisbenzimide (Hoechst) (Figure S3A). To determine if NDR1 regulates the ability of TGFβ to control cell proliferation, we performed BrdU incorporation assays (Figure 4C). As expected, TGFβ markedly reduced the ratio of BrdU-labeled cells, suggesting that TGFβ induces cell cycle arrest in NMuMG cells (Figure 4D). The expression of wild type NDR1, but not kinase-inactive NDR1, opposed TGFβ-suppression of BrdU incorporation in NMuMG cells (Figure 4C, D).

In complementary studies, we found that knockdown of NDR1 enhanced the potency of TGFβ to induce 50% reduction in the population growth of NMuMG cells (EC50) (Figure 4E, F and Figure S3B, C). Interestingly, knockdown of NDR1 together with knockdown of the closely related protein NDR2 further enhanced the potency of TGFβ to induce cell cycle arrest in NMuMG cells (Figure 4E, F, Figure S3C). Collectively, our data suggest that NDR antagonizes the ability of TGFβ to inhibit cell proliferation.

### NDR1 Suppresses TGFβ-dependent Smad2 Phosphorylation

**Figure 5:**
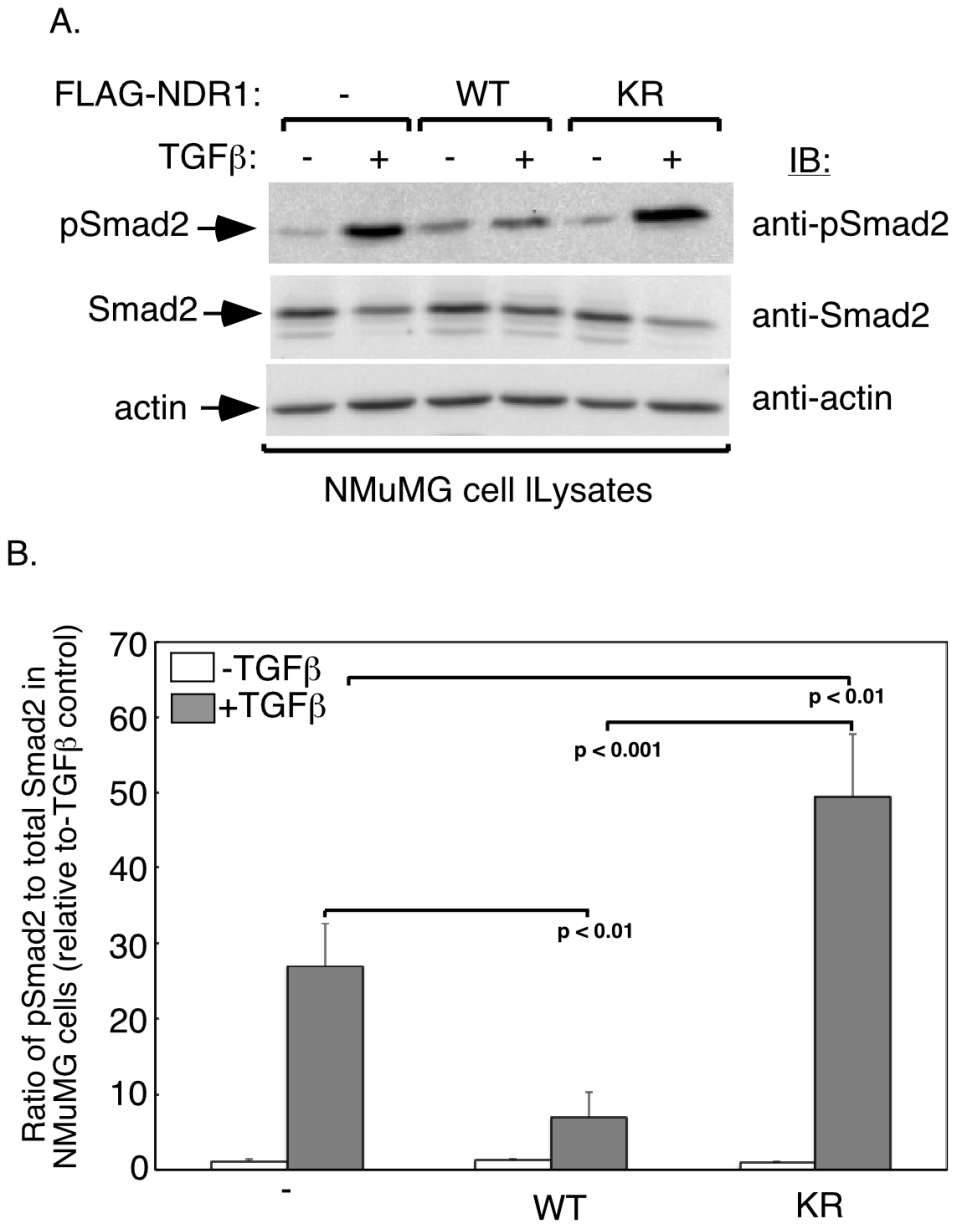
NDR1 impairs TGFβ-phosphorylation of Smad2. **A.** Lysates of untreated or 48h-TGFβ-incubated NMuMG cells transfected with an expression plasmid encoding wild type or kinase-inactive NDR1, or transfected with the vector control were subjected to immunoblotting using an antibody that recognizes Smad2 when phosphorylated specifically at the TGFβ-induced sites (pSmad2) or an antibody that recognizes Smad2 regardless of its phosphorylation status (Smad2) or with an actin antibody, the latter serving as a loading control. **B.** Actin-normalized TGFβ-phosphorylated Smad2 was expressed relative to the actin-normalized total Smad2. Data are presented as the mean+SEM (n = 3) ratio of TGFβ-phosphorylated Smad2 to total Smad2. Statistical significance between TGFβ-induced phosphorylation of Smad2 in the vector control cells and each of the wild type and kinase-inactive NDR1-expressing cells is indicated (ANOVA).

The ability of NDR1 to oppose TGFβ-dependent transcription and cell cycle arrest raised the question of the mechanism by which NDR1 exerts this effect. TGFβ-dependent phosphorylation and consequent nuclear accumulation of the receptor-regulated Smad proteins mediate TGFβ-induced transcription and biological responses. We, therefore, determined the effect of NDR1 on the phosphorylation and nuclear accumulation of Smad2 in NMuMG cells upon exposure to TGFβ. As expected, TGFβ robustly increased the phosphorylation of Smad2 (Figure 5A, B). Strikingly, we found that wild type NDR1 substantially reduced the ability of TGFβ to induce Smad2 phosphorylation in NMuMG cells (Figure 5). Consistent with these results, wild type NDR1 suppressed TGFβ-induced accumulation of Smad2 in the nucleus in NMuMG cells (Figure S4). By contrast to wild type NDR1, expression of the kinase-inactive NDR1 (KR) enhanced TGFβ-induced phosphorylation and nuclear accumulation of Smad2 in NMuMG cells (Fig. 5 and Fig. S4). Together, these data suggest that NDR1 inhibits the ability of TGFβ to trigger the phosphorylation and consequent nuclear accumulation of Smad2 and thereby impairs TGFβ-induced transcription.

### TGFβ Signaling Induces NDR1 Degradation

**Figure 6:**
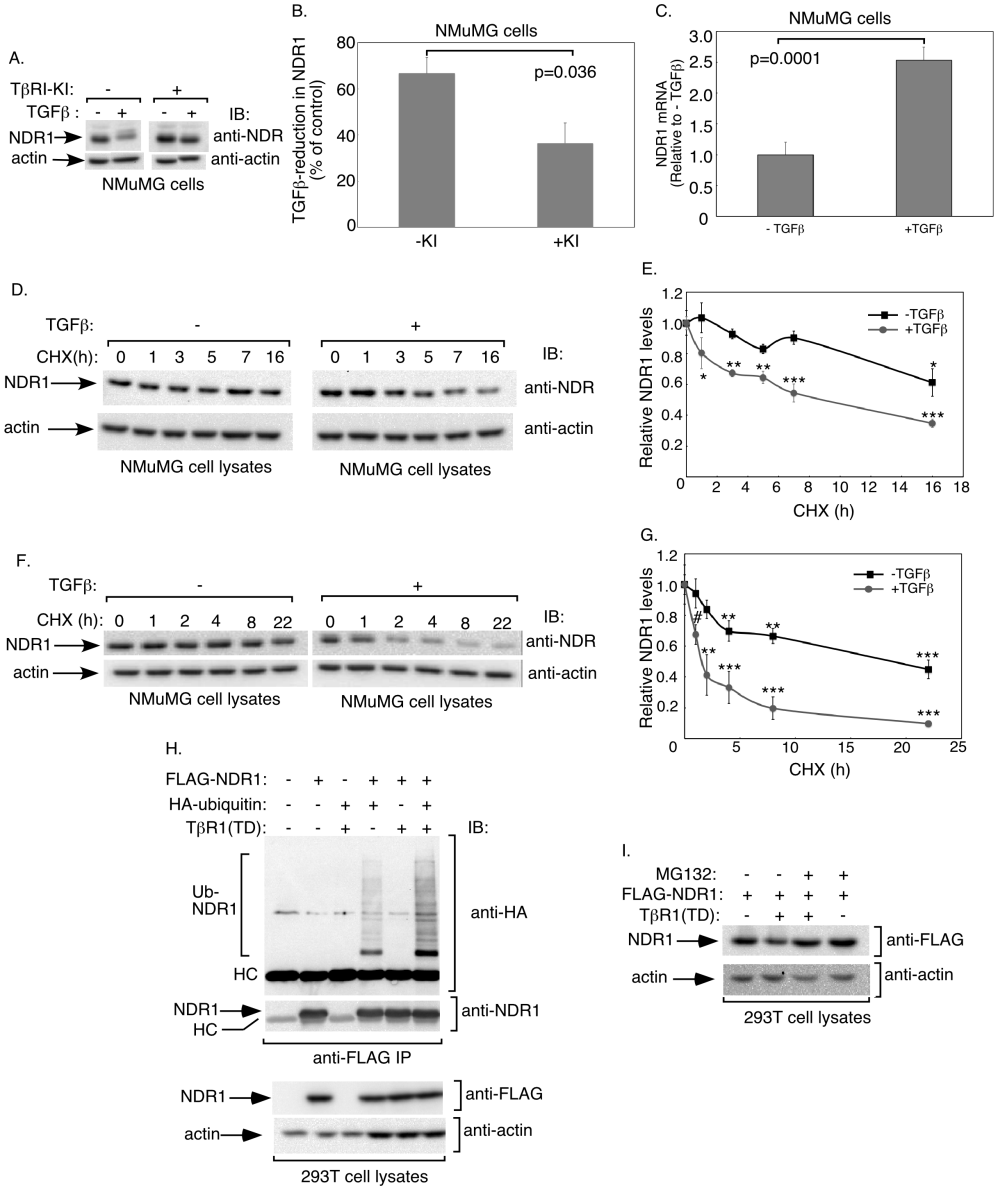
TGFβ signaling promotes NDR1 turnover. **A.** Lysates of untreated or 48h-TGFβ-treated NMuMG cells in the absence or presence of the TGFβ type I receptor kinase inhibitor SB431542 (KI) were subjected to immunoblotting with the NDR1 or actin antibody. **B.** Protein abundance of NDR1 and actin in immunoblots, including those shown in A, were quantified and percent reduction of NDR1 (normalized to actin) in response to TGFβ was analyzed. Data are presented as the mean+SEM (n = 4) of percent decrease in protein abundance of NDR1 in NMuMG cells in response to TGFβ. TGFβ treatment decreased the protein abundance of NDR1 in NMuMG cells. **C.** TGFβ does not repress NDR1 mRNA expression. RNA extracts from untreated or 48h-TGFβ-treated NMuMG cells were analyzed by quantitative RT-PCR for NDR1 and GAPDH mRNA abundance. Data are presented as the mean+SEM (n = 3) of relative mRNA abundance of NDR1 in NMuMG cells. TGFβ did not reduce relative abundance of NDR1 mRNA. Significant differences are indicated in B and C as determined by unpaired, two-tailed t-test. **D.** Lysates of NMuMG cells left untreated or incubated with 10 µg/ml cycloheximide for different times, alone or together with 100 pM TGFβ, were subjected to immunoblotting using the NDR1 or actin antibody. **E.** Protein abundance of NDR1 in immunoblots, including the one shown in Figure 6D, were quantified and normalized to actin. Data are presented as the mean±SEM (n = 3) of normalized protein abundance of NDR1 expressed relative to that at time 0 for the respective minus or plus TGFβ group. Data interpolation indicated that NDR1's half-life was greater than 16 h. TGFβ reduced NDR1's half-life to approximately 9 h. **F.** Lysates of untreated or 24 h-TGFβ-preincubated NMuMG cells followed by exposure to cycloheximide for different time points, were subjected to immunoblotting using NDR1 or actin antibody. **G.** Protein abundance of NDR1 in immunoblots as described and including the one shown in Figure 6F was quantified as described in E. Data are presented as the mean±SEM (n = 4) of relative NDR1 levels. TGFβ reduced the half-life of NDR1 from greater than 16 h to approximately 2.5 h. **H.** TGFβ signaling enhances the ubiquitination of NDR1. Lysates of 293T cells expressing FLAG-NDR1, HA-ubiquitin, and constitutively active TGFβ type I receptor, were subjected to immunoprecipitation using the FLAG antibody, followed by immunoblotting with the HA or NDR1 antibody. Cell lysates were also immunoblotted with the FLAG or actin antibody. HC refers to the heavy chain of the FLAG antibody. I. Lysates of 293T cells transfected with FLAG-NDR1 alone or together with constitutively active TGFβ type I receptor and treated without or with 0.5 µM MG132 (Sigma) for 7 hours were subjected to immunoblotting with the FLAG or actin antibody. *, **, or *** in E and G indicates significant difference from respective control at P<0.05, p<0.01, or p<0.001, respectively (ANOVA). # indicates significant difference from control (p<0.05, unpaired, one tailed t-test).

The identification of NDR1 as a negative regulator of TGFβ-induced transcription and cell cycle arrest raised the important question of whether TGFβ signaling might in turn influence NDR1. We characterized the effect of TGFβ on the abundance of endogenous NDR1 in NMuMG cells. Remarkably, we found that TGFβ stimulation reduced the steady-state levels of NDR1 (Figure 6A, B). Incubation of cells with the TGFβ-type I kinase inhibitor SB431542 (TβRI-KI) restored the abundance of NDR1 protein in TGFβ-treated cells (Figure 6A, B). Using quantitative real-time RT-PCR analyses, we found that TGFβ did not reduce and instead increased the abundance of NDR1 mRNA, suggesting that TGFβ-induced downregulation of NDR1 protein is not due to changes in NDR1 gene expression (Figure 6C).

We next considered the possibility that the downregulation in NDR1 protein in response to TGFβ might result from the increased turnover of NDR1 protein. We measured the rate of NDR1 protein turnover in NMuMG cells treated for different times with the protein synthesis inhibitor cycloheximide in the absence (−) or presence (+) of TGFβ (Figure 6D, E). We found that the half-life of NDR1 in control cells was greater than 16 h, suggesting that NDR1 is a relatively stable protein. Stimulation of cells with TGFβ reduced the half-life of NDR1 to approximately 8 h, suggesting that TGFβ increased the turnover of NDR1 (Figure 6D, E). To further explore the decrease in steady-state levels of NDR1 by long-term activation of TGFβ signaling (Figure 6A, B), we assessed NDR1 turnover rates in cells that were left untreated or pretreated with TGFβ for 24 h prior to the time-course treatment of cycloheximide. Interestingly, we found that pretreatment of cells with TGFβ led to eight-fold reduction in the half-life of NDR1, suggesting that prolonged TGFβ treatment induced substantial degradation of NDR1 (Figure 6F, G). Using in vivo ubiquitination assays, we found that NDR1 was conjugated with ubiquitin in 293T cells (Fig. 6H and Figure S5) [49]. Importantly, expression of a constitutively active TGFβ type I receptor, which activates the Smad signaling pathway in the absence of TGFβ addition [21], robustly stimulated the ubiquitination of NDR1 in cells (Figure 6H and Figure S5A). We also found that exposure of 293T cells to the proteasome inhibitor MG132 suppressed the ability of TGFβ to reduce the abundance of NDR1 (Figure 6I and Figure S5B). Together, these data suggest that TGFβ signaling induces NDR1 ubiquitination and its consequent degradation involving the 26S proteasome (Figure 6H, I and Figure S5A, B).

Collectively, our study identifies an important functional and regulatory link between NDR1 and the TGFβ signaling pathway. NDR1 suppresses TGFβ-induced transcription and cell cycle arrest, and to overcome this effect, TGFβ promotes the ubiquitination and turnover of NDR1.

## Discussion

**Figure 7:**
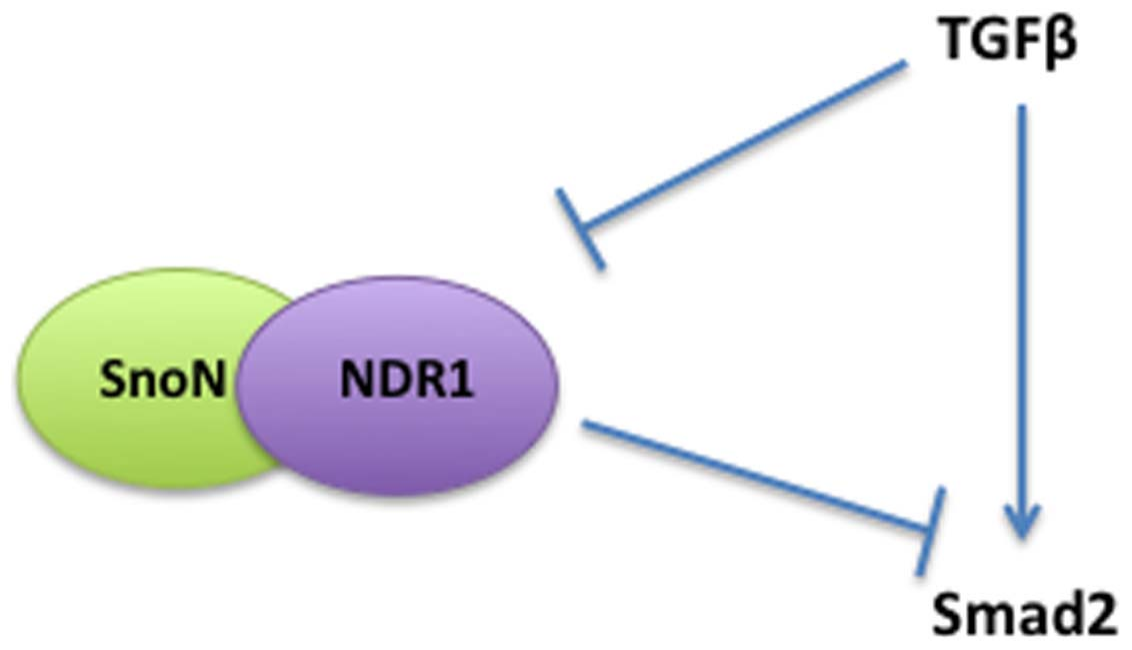
A schematic model showing that SnoN and NDR1 interact, and that NDR1 and TGFβ reciprocally inhibit each other in epithelial cells.

In this study, we have discovered a critical role for the protein kinase NDR1 in the regulation of TGFβ signaling in proliferating cells. We have identified NDR1 as a novel interacting protein with SnoN, a key component of the TGFβ signaling pathway. Loss and gain of function analyses reveal that NDR1 suppresses TGFβ-induced transcription and cell cycle arrest in epithelial cells. NDR1 inhibits the ability of TGFβ to induce the phosphorylation and consequent nuclear accumulation of Smad2, providing the mechanistic basis for NDR1 regulation of TGFβ-induced transcription and cellular responses. Remarkably, we have also found that TGFβ reciprocally regulates NDR1, triggering the degradation of NDR1. These findings define an intimate link between NDR1 and TGFβ signaling, whereby NDR1 inhibits TGFβ-induced transcription and cell cycle arrest, and to counteract this effect, TGFβ enhances the turnover of NDR1 protein (Figure 7).

The finding that NDR1 antagonizes TGFβ-induced cell cycle arrest in epithelial suggests that cancer cells may employ an NDR1-dependent mechanisms to evade the tumor suppressive effect of TGFβ. Consistent with this possibility, we have found that knockdown of NDR1 restores the ability of TGFβ to induce cell cycle arrest in the human breast MDA-MB-231 carcinoma cells, which are resistant to the TGFβ-induced cell cycle arrest (Figure S6A, B, C). Thus, deregulation of NDR1 control of TGFβ signaling may be relevant in cancer pathogenesis.

The identification of NDR1 as a novel regulator of TGFβ-induced transcription advances our understanding of the mechanisms that control TGFβ responses. We have found that NDR1 markedly inhibits TGFβ-induced cell cycle arrest. In future studies, it will be interesting to determine whether NDR1 modulates other TGFβ responses including epithelial-mesenchymal transition, extracellular remodeling, and cell migration, or whether NDR1 specifically regulates cell proliferation.

How does NDR1 inhibit TGFβ-induced transcription and cell cycle arrest? We have found that NDR1 strongly inhibits the phosphorylation and the nuclear accumulation of Smad2. The inhibition of Smad2 phosphorylation provides a basis for NDR1-inhibition of TGFβ-induced transcription and cell cycle arrest. Recent studies suggest that the protein kinase lats, which is related to NDR1, restricts the nuclear accumulation of Smad2 without affecting its phosphorylation [58]. Thus, NDR1 and lats employ distinct mechanisms to regulate TGFβ signaling. How NDR1 inhibits Smad2 phosphorylation remains to be characterized. Since the kinase activity of NDR1 is required for its ability to inhibit Smad2 signaling, it will be critical in future studies to identify substrates of NDR1 that lead to the inhibition of Smad2 phosphorylation.

The finding that TGFβ triggers the degradation of NDR1 proteins suggests that reciprocal negative feedback regulation of NDR1 and TGFβ signaling provides balance in their mutually opposing effects. Intriguingly, TGFβ induces the degradation of SnoN, which as we have found in this study interacts with NDR1. The E3 ubiquitin ligases Cdh1-APC, Smurf2, and Arkadia mediate the TGFβ-induced ubiquitination and consequent degradation of SnoN [49], [50], [59], [60], [61]. In future studies, it will be interesting to determine if these E3 ubiquitin ligases or others induce the ubiquitination of NDR1 in cells upon exposure to TGFβ.

Although we have focused our studies on the identification of NDR1 as a novel regulator of TGFβ signaling in proliferating cells, our findings may have broader implications for both TGFβ signaling and NDR1. In the developing mammalian nervous system, TGFβ-Smad2 signaling has been implicated in the control of axon development, whereby Smad2 inhibits axon growth in granule neurons of the rat cerebellar cortex [62]. In view of our finding demonstrating that NDR1 inhibits Smad2 signaling, it will be interesting to determine whether NDR1 promotes axon growth in mammalian neurons. Conversely, recent studies have revealed that NDR1 controls the development of dendrites and synapses in mouse hippocampal neurons [46]. Our finding that TGFβ induces the degradation of NDR1 raises the interesting question of whether TGFβ might influence these aspects of neuronal morphogenesis.

Our findings have implications beyond cellular development and homeostasis. Since loss of responsiveness to TGFβ-induced cell cycle arrest contributes to tumorigenesis [10], [11], [12], [13], [14], the identification of a novel role for NDR1 in TGFβ signaling suggests that NDR1 may also influence tumor initiation. Notably, NDR1 is upregulated in lung and mammary carcinomas [47], [48], raising the possibility that NDR1 might contribute to loss of TGFβ responsiveness in these tumors. Thus, our study raises the potential for NDR1 as a target for drug discovery in cancer biology.

## Materials and Methods

### Plasmids

A pCaGiP vector was used to generate FLAG, HA double epitope-tagged SnoN or FLAG epitope-tagged NDR1 stable expression constructs, where a bicistronic transcript containing an internal ribosomal entry site (IRES) encoded the puromycin resistance marker and the protein of interest [39], [63], [64]. FLAG, HA-tagged SnoN containing nucleotides to express FLAG, Tobacco Etched Virus (TEV) protease site (ENLYFQG), and HA peptides upstream of the SnoN cDNA was generated using a polymerase chain reaction (PCR)-based cloning approach. Expression vectors to express fusion proteins of *Renillla* luciferase (Rluc) with wild type or deletion mutant SnoN were generated by PCR-based amplification and subcloning of the Rluc cDNA upstream of SnoN cDNA in CMV-based (pCMV5B) SnoN-expression vectors [37], [49], [50], [65]. The NDR1 cDNA product of PCR amplification of epithelial cell-derived polyA-cDNA using NDR1gene-specific oligonucleotides was used to generate HA- and FLAG-tagged NDR1 expression vectors (pCMV5B and pCaGiP). Constructs expressing Rluc in fusion with wild type or deletion mutant NDR1 were generated as described for Rluc-SnoN. NDR1 and NDR2 RNA interference (RNAi) plasmids were constructed using the pU6/CMV/enhanced green fluorescent protein (GFP) expression control vector, with NDR1 or NDR2 shorthairpin RNAs (shRNAs), and GFP under the control of U6 and CMV promoters, respectively [40]. Two shRNAs-expressing constructs were generated to target distinct regions in each of NDR1 and NDR2 mRNAs as follows: NDR1i-1, 5′GCAACCTTATCGCTCAACAT3′, NDR1i-2, 5′GGCAGACAGTTTGTGGGT TGT3′, NDR2i-1, 5′GGAGGTGACATGATGACATT3′, and NDR2i-2, 5′GCAGACTG GTTACAACAAATT3′. All constructs were verified by restriction digests and DNA sequencing analysis (University of Calgary Core Sequencing Facility).

### Cell Cultures and Transfections

The human keratinocyte HaCaT [66], embryonic kidney 293T [67], [68], and mouse mammary gland epithelial NMuMG [69], [70], [71] cells were obtained from ATCC and maintained in appropriate growth conditions [39], [40], [63], [72]. The human breast carcinoma MDA-MB-231 cells, a generous gift from Dr. Frank Jirik, were maintained in Dulbecco's Modified Eagle Medium containing 10% fetal bovine serum [73], [74]. 293T cells were transfected using the calcium phosphate method [40]. HaCaT cells were transiently transfected using *Fugene* (Roche), *Trans*IT-LT1 reagent (Mirus), or Lipofectamine-LTX (Invitrogen) according to the manufacturer’s instructions. HaCaT cells were transfected with the pCAGiP vector (vector control) or one encoding the FLAG, HA-SnoN using Lipofectin (Invitrogen) and incubation in 0.45 µg/ml puromycin (Invitrogen)-containing medium for selection of control vector or SnoN stably expressing cells. A similar strategy was used to generate control and FLAG-NDR1 expressing NMuMG cells with the exception of using 2 µg/ml puromycin. MDA-MB-231 were transfected using Lipofectamin and plus reagents (Invitrogen).

### Immunoprecipitations, Immunoblottings, and TAP

Cells were lysed in TNE-based buffer-containing 0.5% Triton X-100, protease and phosphatase inhibitors, and lysates were cleared by centrifugation and subjected to protein concentration determination using the Bradford protein assay (BioRad) [39], [40], [63], [75]. For interaction studies, equivalent amounts of protein, representing a maximum of approximately 90% of total protein content per sample, were subjected to immunoprecipitations using appropriate antibodies. Immunoprecipitates and aliquots of the lysates were subjected to immunoblottings or *Renilla* luciferase assays using the *Renilla* luciferase kit (Promega) and the Orion II luminometer (Berthold Detection Systems) detection system. Immunoprecipitates-containing Rluc-fusion proteins (or Rluc protein alone) were resuspended in TNE buffer containing 0.1% Triton X-100 prior to measuring any associated luciferase activity [53]. Antibodies used in the immunoprecipitations and immunoblottings included rabbit anti-SnoN (H317, Santa Cruz), mouse anti-Flag (M2, Sigma), mouse anti-Myc (9E10, Covance), mouse anti-HA (16B12, Covance), rabbit anti-Actin (Sigma), mouse anti-NDR1 (2G8–1F3, Abnova), rabbit anti-NDR1 (H100, Santa Cruz), mouse anti-Smad2/3 (BD-Transduction laboratories), rabbit anti-phospho (Ser465/467) Smad2 (Calbiochem), and horseradish peroxidase-conjugated anti-mouse and anti-rabbit IgG antibodies (GE healthcare). Immunoblotting-generated enhanced chemiluminescence signals were visualized and quantified using a Versadoc 5000 Imager (Bio-Rad) and Quantity One software, respectively [39], [40], [63], [75]. For tandem affinity purification (TAP) experiments, lysates of FLAG, HA-SnoN-expressing HaCaT cells or vector control-transfected HaCaT cells were subjected to immunoprecipitation using anti-FLAG® M2 antibody affinity gel (Sigma), elution of immunocomplexes by treatment with TEV enzyme, immunoprecipitation of the eluate with anti-HA affinity matrix (Roche), and partial separation of immunoprecipitates by SDS-PAGE. Processing and analysis of colloidal Coomassie-stained gel slices was performed at the Southern Alberta Mass Spectrometry (SAMS) Centre for Proteomics by LC-MS/MS [51], [52].

### Luciferase Reporter Assays

HaCaT and NMuMG cells were seeded at 2.5 to 3.5×10^4^ cells/well in 24-well plates. Cells were co-transfected with the 3TP-*Firefly* luciferase reporter constructs, the CMV-β-galactosidase or pR-TK *Renilla* luciferase internal control reporter constructs, together with a control vector or one encoding an NDR1 protein, or with a control or NDR1 RNAi vector, incubated for 16 to 18 h in 0.2% fetal bovine serum-containing medium in the absence or presence of 100 pM TGFβ (R & D Systems, Minneapolis, MN), lysed and subjected to single or dual luciferase activity assays [39], [40], [63], [72], [75], with each experimental condition carried out in triplicates. Each replicate's arbitrary *Firefly* luciferase activity, in relative light units, was normalized to its β-galactosidase or *Renilla*-luciferase activity, to control for variations in transfection efficiency.

### Quantitative Real Time PCR

DNase-treated TRIzol (Gibco)-extracted RNA from NMuMG cells cultured in the absence or presence TGFβ was reverse transcribed using SuperScript II transcriptase (Invitrogen) and oligo-(dT)_12–18_ (Amersham Biosciences) [40], [63], [75], [76]. The polyA-cDNAs were subjected to quantitative PCR using gene-specific primers for Plasminogen Activator Inhibitor 1 (forward-5′TCTCAGAGGTGGAAAGAGCCAG3′, reverse-5′TGAAGTAGAGGGCATTCACCAGC3′), NDR1 (forward-5′ATTTGGTGAGGTACGGCTTG3′ and reverse-5′CAGGCAGGAACTCCA TGATT3′), and the house-keeping gene glyceraldehyde-3-phosphate dehydrogenase (forward-5′TCAACAGCAACTCCCACTCTTCCA3′ and reverse-5′ACCCTGTTGCTGTAGCCGTATTC A3′) using a 2X Sybr Green Mix (BioRad) and Rotor-Gene Thermocycler (Corbett Research). The specificity of the products was confirmed using the melt curve method. Data were analyzed and expressed as described [75].

### Microscopy and Cell Proliferation Assays

For fluorescence microscopy experiments, NMuMG cells left untreated or incubated with TGFβ for two days were formaldehyde-fixed, and incubated with DNA dye bisbenzimide (Hoechst) (Invitrogen). For indirect Smad2/3 immunofluorescence, untreated or TGFβ-treated NMuMG cells were incubated with a mouse Smad2/3 antibody, and a Cy3-labelled anti-mouse antibody (Jackson Laboratories) in the presence of the DNA dye Hoechst. Images of cells were captured using a Kinetic Scan Reader (KSR) (Cellomics, Inc., Pittsburgh, PA) equipped with a Carl Zeiss Axiom x microscope and a charge-coupled device (CCD) digital camera [39], [63]. Bromodeoxyuridine (BrdU) assays were carried out using a BrdU assay kit (Roche), fluorescence images were captured using fluorescence microscopy, and data were generated using the Target Activation Bio-Application of the Cellomics KSR [39]. For cell growth rates analyses, cells were grown for one, two or three days in the presence or absence of 100 pM TGFβ, and counted using a haemocytometer prior to and at each day during treatment. Alternatively, population cell growth was determined based on nuclei counts in fixed cells stained with the DNA dye Hoechst. Cell counts were normalized to cell numbers before treatment, and replicate values were averaged. For RNAi assays, NMuMG or MDA-MB-231 cells were transfected with GFP-expressing plasmids containing an RNAi control vector, or the NDR1 shRNAs alone or together with NDR2 shRNA expressing vectors as described in the legends of Figure 4, Figure S3, and Figure S6. Untreated or TGFβ-incubated NMuMG or MDA-MB-231 cells were fixed and incubated with the DNA dye Hoechst 36 h after ligand treatment. Cells were identified by GFP (green) and nuclei (Hoechst) (blue) fluorescence signals, and the number of GFP-positive cells was assayed using the Target Activation Bio-Application of the Cellomics KSR instrument. Data were analyzed as described in figure legends.

### Statistical Analysis

Data were subjected to Analysis of Variance (ANOV) or student t-test as indicated in the figure legends with significant difference set at p<0.05.

## Supporting Information

Figure S1 **Related to** **Figure 1** **.** **A.** Lysates of 293T cells expressing *Renilla* luciferase (Rluc), alone, or as fusion with SnoN (Rluc-SnoN) were subjected to immunoprecipitation using the NDR1 antibody or IgG immunoglobulins, as a negative control, followed by analysis of immunoprecipitates by luciferase assays (90%) or immunoblotting (10%) with NDR1 antibody (data not shown). Cell lysates were also analyzed by luciferase assays and immunoblotting using NDR1 or actin antibody (data not shown). Endogenous NDR1-associated Rluc or Rluc-SnoN luciferase (IgG-subtracted) were normalized to Rluc or Rluc-SnoN, respectively, and endogenous NDR1 expression. The data are presented as the mean +SEM (n = 3) of NDR1-associated Rluc activity relative to Rluc activity associated with NDR1 in the case of the Rluc control. Rluc-SnoN associated robustly with endogenous NDR1. **B.** Lysates of 293T cells expressing Rluc or Rluc-NDR1 were subjected to immunoprecipitation using a SnoN antibody or IgG immunoglobulins, as a negative control, followed by analysis of the immunoprecipitates by luciferase assays (90%) or immunoblotting (10%) with SnoN antibody (data not shown). Cell lysates were also subjected to luciferase assays or immunoblotting with SnoN or actin antibody (data not shown). Endogenous SnoN-associated Rluc or Rluc-NDR1 activity was determined as in A. Data are presented as the mean+SEM (n = 4) of SnoN-associated Rluc activity relative to Rluc activity associated with SnoN in the case of the Rluc control. Rluc-NDR1 interacted strongly with endogenous SnoN. **C.** Lysates of untreated or TGFβ-treated 293T cells were subjected to immunoprecipitation using NDR1 antibody or IgG immunoglobulins, as a negative control, followed by immunoblotting with the SnoN or NDR1 antibody. Cell lysates were also subjected to immunoblotting with the SnoN, NDR1 or actin antibody with the latter serving as a loading control. **** in A and B indicates significant difference from the control (p<0.0001, t-test).
(TIF)

Figure S2 **Related to** **Figure 2** **.** **A.** Lysates of 293T cells expressing HA-NDR1 in the presence of the control RNAi vector, or NDR1 RNAi NDR1i-1 or NDR1i-2 plasmid were subjected to immunoblotting using the HA or actin antibody, with the latter to serve as a loading control. NDR1i-1 or NDR1i-2 induced 80 to 90 percent knockdown of NDR1. **B.** Lysates of NMuMG cells transfected with increasing concentrations of a plasmid expressing HA-NDR1 together with the TGFβ-responsive 3TP-luciferase reporter and a transfection efficiency vector as described in Figure 2C, were subjected to immunoblotting using the HA or actin antibody. Images in A and B are representative blots from experiments that were repeated at least two independent times.
(TIF)

Figure S3 **Related to** **Figure 4** **.** **A.** Population growth of NMuMG cells expressing wild type (WT) or kinase-inactive (KR) NDR1, or control vector (−) after culturing for one, two, or three days in the absence or presence of 100 pM TGFβ was determined by subjecting DNA dye (Hoechst)-labeled NMuMG cells to fluorescence microscopy and data analysis using the Cellomics KSR platform and Target Activation algorithm. Percent decrease in population growth of NMuMG cells by TGFβ was quantified as described in Figure 4B. Data are presented as the mean+SEM of percent reduction of population growth of NMuMG cells by TGFβ from three (day 1 and day 3) or five (day 2) independent experiments. ** or *** indicates significant difference from the respective control within each day at p<0.01, or P<0.001, respectively (ANOVA). **B.** Representative fluorescence images of NMuMG cells one day post transfection with control RNAi, NDR1i or NDRi plasmids as described Figure 4E, where the DNA dye Hoechst (blue) and GFP (green)-induced signals indicate total NMuMG cells and transfected NMuMG cells, respectively. Analysis of the GFP-labeled cells as compared to total cells using the target activation algorithm indicated approximately 50 percent transfection efficiency for all three sets of transfections. The width of each micrograph corresponds to 330 µm. **C.** For each experiment including the one shown in Figure 4E, triplicate average of GFP-positive cells at each TGFβ concentration was determined. Data are presented as the mean±SEM of relative GFP-positive cell numbers from six (control and NDRi) or five (NDR1i) independent experiments.
(TIF)

Figure S4 **Related to** **Figure 5** **.** Representative images of untreated or TGFβ-treated NMuMG cells expressing wild type or kinase-inactive NDR1 or vector control that were subjected to indirect immunofluorescence using the Smad2 antibody and a Cy3-secondary antibody (red) and labeling with the DNA Hoechst dye (blue), and scanned by fluorescence microscopy. The width of each micrograph corresponds to 330 µm.
(TIF)

Figure S5 **Related to** **Figure 6** **.** A. Lysates of 293T cells coexpressing FLAG-NDR1 and HA-ubiquitin alone or together with the constitutively active TGFβ type I receptor, harboring a mutation in Threonine 204 to aspartate, were subjected to immunoprecipitation using the FLAG antibody followed by immunoblotting with the HA or NDR1 antibody as described in Figure 6H. Ubiquitin-conjugated NDR1 protein species as indicated in and including the protein species in Figure 6H immunoblots were quantified and normalized to NDR1 levels in the immunoprecipitates. Data are presented as the mean+SEM (n = 3) of ubiquitin-conjugated NDR1 species relative to the ubiquitinated NDR1 in cells coexpressing NDR1 and ubiquitin. Significant difference between the two groups was determined using unpaired, two-tailed t-test. B. Lysates of untreated or MG132-treated 293T cells expressing FLAG-NDR1 alone or together with constitutively active receptor were subjected to FLAG and actin immunoblotting as described in Figure 6I. NDR1 protein species as indicated and including the protein species in Figure 6I were quantified and normalized to respective actin. Data are presented as the mean+SEM (n = 6) of NDR1 relative to NDR1 in cells expressing NDR1 alone and left in the absence of MG132. *** indicates significant difference from the control (p<0.001, ANOVA).
(TIF)

Figure S6 **NDR1 knockdown restores the ability of TGFβ to inhibit cell proliferation in the breast MDA-MB-231 carcinoma cells.** A. Lysates of MDA-MB-231 transfected with a control or NDR1 RNAi plasmids, were subjected to immunoblotting with an NDR1 or actin antibody. Values shown below lanes 1 and 2 represent actin-normalized NDR1 level expressed relative to the actin-normalized NDR1 level in the RNAi control vector transfected cells. B. GFP-expressing and DNA-Hoechst-labeled MDA-MB-231 cells transfected as in A and incubated one day post transfection with 0, 25, 100, or 400 pM TGFβ for 72 h were imaged and quantified by fluorescence microscopy and the target activation bio-application, respectively, using the Cellomics KSR as in Figure 4 and Figure S3. Untreated or TGFβ-treated cells were seeded in triplicates or quadruplicates in a 96-well plate, and population growth of GFP-positive cells were averaged. Data are presented as the mean+SEM of average population growth of GFP-positive MDA-MB-231 cells from five independent experiments expressed relative to the untreated control. C. MDA-MB-231 cells transfected as in A and left untreated or treated with 400 pM TGFβ were incubated for the last hour with bromodeoxyuridine, and subjected to immunocytochemistry using a BrdU antibody, fluorescence microscopy and analysis as described in Figure 4. Target activation bioapplication was used to quantify ratio of GFP-expressing BrdU labeled cells, and averages of replicates quantified as in B. Data are presented as the mean+SEM of GFP-expressing BrdU-positive cells from 5 independent experiments. ** or *** indicates significant difference from the control at p<0.01, or p<0.001, respectively (ANOVA).
(TIF)

## References

[1] Massague J (1990) The transforming growth factor-beta family. Annu Rev Cell Biol 6: 597–641. 2177343 10.1146/annurev.cb.06.110190.003121

[2] Roberts AB, Sporn MB (1990) The transforming growth factor-betas. In: Sporn MB, Roberts AB, editors. Peptide growth factors and their receptors. Heidelberg: Springer-Verlag. 419–472.

[3] Sporn MB, Roberts AB (1990) TGF-beta: problems and prospects. Cell Regul 12: 875–882. 10.1091/mbc.1.12.875 PMC362858 2100192

[4] Wu MY, Hill CS (2009) Tgf-beta superfamily signaling in embryonic development and homeostasis. Dev Cell 16: 329–343. 19289080 10.1016/j.devcel.2009.02.012

[5] Blobe GC, Schiemann WP, Lodish HF (2000) Role of transforming growth factor beta in human disease. N Engl J Med 342: 1350–1358. 10793168 10.1056/NEJM200005043421807

[6] Lan HY (2011) Diverse roles of TGF-beta/Smads in renal fibrosis and inflammation. Int J Biol Sci 7: 1056–1067. 21927575 10.7150/ijbs.7.1056 PMC3174390

[7] Schmierer B, Hill CS (2007) TGFbeta-SMAD signal transduction: molecular specificity and functional flexibility. Nat Rev Mol Cell Biol 8: 970–982. 18000526 10.1038/nrm2297

[8] Tatler AL, Jenkins G (2012) TGF-beta Activation and Lung Fibrosis. Proc Am Thorac Soc 9: 130–136. 22802287 10.1513/pats.201201-003AW

[9] Iwano M (2010) EMT and TGF-beta in renal fibrosis. Front Biosci (Schol Ed) 2: 229–238. 20036943 10.2741/s60

[10] Galliher AJ, Neil JR, Schiemann WP (2006) Role of transforming growth factor-beta in cancer progression. Future Oncol 2: 743–763. 17155901 10.2217/14796694.2.6.743

[11] Kim SJ, Letterio J (2003) Transforming growth factor-beta signaling in normal and malignant hematopoiesis. Leukemia 17: 1731–1737. 12970772 10.1038/sj.leu.2403069

[12] Massague J, Blain SW, Lo RS (2000) TGFbeta signaling in growth control, cancer, and heritable disorders. Cell 103: 295–309. 11057902 10.1016/s0092-8674(00)00121-5

[13] Rahimi RA, Leof EB (2007) TGF-beta signaling: a tale of two responses. J Cell Biochem 102: 593–608. 17729308 10.1002/jcb.21501

[14] Meulmeester E, Ten Dijke P (2010) The dynamic roles of TGF-beta in cancer. J Pathol 223: 205–218. 20957627 10.1002/path.2785

[15] Attisano L, Wrana JL, Lopez-Casillas F, Massague J (1994) TGF-beta receptors and actions. Biochim Biophys Acta 1222: 71–80. 8186268 10.1016/0167-4889(94)90026-4

[16] Huang T, David L, Mendoza V, Yang Y, Villarreal M, et al (2011) TGF-beta signalling is mediated by two autonomously functioning TbetaRI:TbetaRII pairs. EMBO J 30: 1263–1276. 21423151 10.1038/emboj.2011.54 PMC3094126

[17] Massague J, Attisano L, Wrana JL (1994) The TGF-beta family and its composite receptors. Trends Cell Biol 4: 172–178. 14731645 10.1016/0962-8924(94)90202-x

[18] Wrana JL, Carcamo J, Attisano L, Cheifetz S, Zentella A, et al.. (1992) The type II TGF-β Receptor Signals Diverse Responses in Co-Operation with the Type I Receptor. Cold Spring Harbour Symposia on Quantitative Biology. 81–86. 10.1101/sqb.1992.057.01.011 1339707

[19] Wrana JL, Tran H, Attisano L, Arora K, Childs SR, et al (1994) Two distinct transmembrane serine/threonine kinases from Drosophila form an activin receptor complex. Mol Cell Biol 14: 944–950. 8289834 10.1128/mcb.14.2.944-950.1994 PMC358449

[20] Miyazono K, Suzuki H, Imamura T (2003) Regulation of TGF-beta signaling and its roles in progression of tumors. Cancer Sci 94: 230–234. 12824914 10.1111/j.1349-7006.2003.tb01425.x PMC11160178

[21] Wieser R, Wrana JL, Massague J (1995) GS domain mutations that constitutively activate TβR-I, the downstream signalling component in the TGF-β receptor complex. EMBO J 14: 2199–2208. 7774578 10.1002/j.1460-2075.1995.tb07214.x PMC398326

[22] Wrana JL, Attisano L, Wieser R, Ventura F, Massague J (1994) Mechanism of activation of the TGF–β receptor. Nature 370: 341–347. 8047140 10.1038/370341a0

[23] Massague J, Wotton D (2000) Transcriptional control by the TGF-beta/Smad signaling system. Embo J 19: 1745–1754. 10775259 10.1093/emboj/19.8.1745 PMC302010

[24] Shi Y, Massague J (2003) Mechanisms of TGF-beta signaling from cell membrane to the nucleus. Cell 113: 685–700. 12809600 10.1016/s0092-8674(03)00432-x

[25] Abdollah S, Macias-Silva M, Tsukazaki T, Hayashi H, Attisano L, et al (1997) TbetaRI phosphorylation of Smad2 on Ser465 and Ser467 is required for Smad2-Smad4 complex formation and signaling. J Biol Chem 272: 27678–27685. 9346908 10.1074/jbc.272.44.27678

[26] Souchelnytskyi S, Tamaki K, Engstrom U, Wernstedt C, ten Dijke P, et al (1997) Phosphorylation of Ser465 and Ser467 in the C terminus of Smad2 mediates interaction with Smad4 and is required for transforming growth factor-beta signaling. J Biol Chem 272: 28107–28115. 9346966 10.1074/jbc.272.44.28107

[27] Lagna G, Hata A, Hemmati-Brivanlou A, Massagué J (1996) Partnership between DPC4 and SMAD proteins in TGF- signalling pathways. Nature 383: 832–836. 8893010 10.1038/383832a0

[28] Lonn P, Moren A, Raja E, Dahl M, Moustakas A (2009) Regulating the stability of TGFbeta receptors and Smads. Cell Res 19: 21–35. 19030025 10.1038/cr.2008.308

[29] Feng XH, Derynck R (2005) Specificity and Versatility in TGF- Signaling Through Smads. Annu Rev Cell Dev Biol. 10.1146/annurev.cellbio.21.022404.142018 16212511

[30] Wotton D, Massague J (2001) Smad transcriptional corepressors in TGF beta family signaling. Curr Top Microbiol Immunol 254: 145–164. 11190572

[31] Wrana JL, Attisano L (2000) The Smad pathway. Cyto Growth Factor Rev in press. 10.1016/s1359-6101(99)00024-6 10708948

[32] Luo K (2004) Ski and SnoN: negative regulators of TGF-beta signaling. Curr Opin Genet Dev 14: 65–70. 15108807 10.1016/j.gde.2003.11.003

[33] Pot I, Bonni S (2008) SnoN in TGF-beta signaling and cancer biology. Curr Mol Med 8: 319–328. 18537639 10.2174/156652408784533797

[34] Pot I, Ikeuchi Y, Bonni A, Bonni S (2010) SnoN: bridging neurobiology and cancer biology. Curr Mol Med 10: 667–673. 20712586 10.2174/156652410792630616 PMC3064562

[35] Bonni S, Bonni A (2012) SnoN signaling in proliferating cells and postmitotic neurons. FEBS Lett 586: 1977–1983. 22710173 10.1016/j.febslet.2012.02.048 PMC3383335

[36] Deheuninck J, Luo K (2009) Ski and SnoN, potent negative regulators of TGF-beta signaling. Cell Res 19: 47–57. 19114989 10.1038/cr.2008.324 PMC3103856

[37] Stroschein SL, Wang W, Zhou S, Zhou Q, Luo K (1999) Negative feedback regulation of TGF-beta signaling by the SnoN oncoprotein. Science 286: 771–774. 10531062 10.1126/science.286.5440.771

[38] Sun Y, Liu X, Ng-Eaton E, Lodish HF, Weinberg RA (1999) SnoN and Ski protooncoproteins are rapidly degraded in response to transforming growth factor beta signaling. Proc Natl Acad Sci U S A 96: 12442–12447. 10535941 10.1073/pnas.96.22.12442 PMC22943

[39] Sarker KP, Kataoka H, Chan A, Netherton SJ, Pot I, et al (2008) ING2 as a novel mediator of transforming growth factor-beta-dependent responses in epithelial cells. J Biol Chem 283: 13269–13279. 18334480 10.1074/jbc.M708834200 PMC2442333

[40] Sarker KP, Wilson SM, Bonni S (2005) SnoN is a cell type-specific mediator of transforming growth factor-beta responses. J Biol Chem 280: 13037–13046. 15677458 10.1074/jbc.M409367200

[41] Hergovich A, Stegert MR, Schmitz D, Hemmings BA (2006) NDR kinases regulate essential cell processes from yeast to humans. Nat Rev Mol Cell Biol 7: 253–264. 16607288 10.1038/nrm1891

[42] Cornils H, Kohler RS, Hergovich A, Hemmings BA (2011) Downstream of human NDR kinases: impacting on c-myc and p21 protein stability to control cell cycle progression. Cell Cycle 10: 1897–1904. 21593588 10.4161/cc.10.12.15826

[43] Cornils H, Kohler RS, Hergovich A, Hemmings BA (2011) Human NDR kinases control G(1)/S cell cycle transition by directly regulating p21 stability. Mol Cell Biol 31: 1382–1395. 21262772 10.1128/MCB.01216-10 PMC3135299

[44] Cornils H, Stegert MR, Hergovich A, Hynx D, Schmitz D, et al (2010) Ablation of the kinase NDR1 predisposes mice to the development of T cell lymphoma. Sci Signal 3: ra47. 20551432 10.1126/scisignal.2000681

[45] Vichalkovski A, Gresko E, Cornils H, Hergovich A, Schmitz D, et al (2008) NDR kinase is activated by RASSF1A/MST1 in response to Fas receptor stimulation and promotes apoptosis. Curr Biol 18: 1889–1895. 19062280 10.1016/j.cub.2008.10.060

[46] Ultanir SK, Hertz NT, Li G, Ge WP, Burlingame AL, et al (2012) Chemical genetic identification of NDR1/2 kinase substrates AAK1 and Rabin8 Uncovers their roles in dendrite arborization and spine development. Neuron 73: 1127–1142. 22445341 10.1016/j.neuron.2012.01.019 PMC3333840

[47] Adeyinka A, Emberley E, Niu Y, Snell L, Murphy LC, et al (2002) Analysis of gene expression in ductal carcinoma in situ of the breast. Clin Cancer Res 8: 3788–3795. 12473591

[48] Hergovich A, Cornils H, Hemmings BA (2008) Mammalian NDR protein kinases: from regulation to a role in centrosome duplication. Biochim Biophys Acta 1784: 3–15. 17881309 10.1016/j.bbapap.2007.07.017

[49] Bonni S, Wang HR, Causing CG, Kavsak P, Stroschein SL, et al (2001) TGF-beta induces assembly of a Smad2-Smurf2 ubiquitin ligase complex that targets SnoN for degradation. Nat Cell Biol 3: 587–595. 11389444 10.1038/35078562

[50] Stroschein SL, Bonni S, Wrana JL, Luo K (2001) Smad3 recruits the anaphase-promoting complex for ubiquitination and degradation of SnoN. Genes Dev 15: 2822–2836. 11691834 10.1101/gad.912901 PMC312804

[51] Nakatani Y, Ogryzko V (2003) Immunoaffinity purification of mammalian protein complexes. Methods Enzymol 370: 430–444. 14712665 10.1016/S0076-6879(03)70037-8

[52] Puig O, Caspary F, Rigaut G, Rutz B, Bouveret E, et al (2001) The tandem affinity purification (TAP) method: a general procedure of protein complex purification. Methods 24: 218–229. 11403571 10.1006/meth.2001.1183

[53] Barrios-Rodiles M, Brown KR, Ozdamar B, Bose R, Liu Z, et al (2005) High-throughput mapping of a dynamic signaling network in mammalian cells. Science 307: 1621–1625. 15761153 10.1126/science.1105776

[54] Kortlever RM, Nijwening JH, Bernards R (2008) Transforming growth factor-beta requires its target plasminogen activator inhibitor-1 for cytostatic activity. J Biol Chem 283: 24308–24313. 18614541 10.1074/jbc.M803341200 PMC3259838

[55] Wilkins-Port CE, Ye Q, Mazurkiewicz JE, Higgins PJ (2009) TGF-beta1+ EGF-initiated invasive potential in transformed human keratinocytes is coupled to a plasmin/MMP-10/MMP-1-dependent collagen remodeling axis: role for PAI-1. Cancer Res 69: 4081–4091. 19383899 10.1158/0008-5472.CAN-09-0043 PMC2962982

[56] Wrana JL, Attisano L, Carcamo J, Zentella A, Doody J, et al (1992) TGF beta signals through a heteromeric protein kinase receptor complex. Cell 71: 1003–1014. 1333888 10.1016/0092-8674(92)90395-s

[57] Miettinen PJ, Ebner R, Lopez AR, Derynck R (1994) TGF-beta induced transdifferentiation of mammary epithelial cells to mesenchymal cells: involvement of type I receptors. J Cell Biol 127: 2021–2036. 7806579 10.1083/jcb.127.6.2021 PMC2120317

[58] Varelas X, Samavarchi-Tehrani P, Narimatsu M, Weiss A, Cockburn K, et al (2010) The Crumbs complex couples cell density sensing to Hippo-dependent control of the TGF-beta-SMAD pathway. Dev Cell 19: 831–844. 21145499 10.1016/j.devcel.2010.11.012

[59] Levy L, Howell M, Das D, Harkin S, Episkopou V, et al (2007) Arkadia activates Smad3/Smad4-dependent transcription by triggering signal-induced SnoN degradation. Mol Cell Biol 27: 6068–6083. 17591695 10.1128/MCB.00664-07 PMC1952153

[60] Nagano Y, Mavrakis KJ, Lee KL, Fujii T, Koinuma D, et al (2007) Arkadia induces degradation of SnoN and c-Ski to enhance transforming growth factor-beta signaling. J Biol Chem 282: 20492–20501. 17510063 10.1074/jbc.M701294200

[61] Wan Y, Liu X, Kirschner MW (2001) The anaphase-promoting complex mediates TGF-beta signaling by targeting SnoN for destruction. Mol Cell 8: 1027–1039. 11741538 10.1016/s1097-2765(01)00382-3

[62] Stegmuller J, Huynh MA, Yuan Z, Konishi Y, Bonni A (2008) TGFbeta-Smad2 signaling regulates the Cdh1-APC/SnoN pathway of axonal morphogenesis. J Neurosci 28: 1961–1969. 18287512 10.1523/JNEUROSCI.3061-07.2008 PMC6671436

[63] Netherton SJ, Bonni S (2010) Suppression of TGFbeta-induced epithelial-mesenchymal transition like phenotype by a PIAS1 regulated sumoylation pathway in NMuMG epithelial cells. PLoS One 5: e13971. 21103059 10.1371/journal.pone.0013971 PMC2980481

[64] von Both I, Silvestri C, Erdemir T, Lickert H, Walls JR, et al (2004) Foxh1 is essential for development of the anterior heart field. Dev Cell 7: 331–345. 15363409 10.1016/j.devcel.2004.07.023

[65] Huynh MA, Ikeuchi Y, Netherton S, de la Torre-Ubieta L, Kanadia R, et al (2011) An isoform-specific SnoN1-FOXO1 repressor complex controls neuronal morphogenesis and positioning in the mammalian brain. Neuron 69: 930–944. 21382553 10.1016/j.neuron.2011.02.008 PMC3073069

[66] Boukamp P, Petrussevska RT, Breitkreutz D, Hornung J, Markham A, et al (1988) Normal keratinization in a spontaneously immortalized aneuploid human keratinocyte cell line. J Cell Biol 106: 761–771. 2450098 10.1083/jcb.106.3.761 PMC2115116

[67] DuBridge RB, Tang P, Hsia HC, Leong PM, Miller JH, et al (1987) Analysis of mutation in human cells by using an Epstein-Barr virus shuttle system. Mol Cell Biol 7: 379–387. 3031469 10.1128/mcb.7.1.379-387.1987 PMC365079

[68] Heiz M, Grunberg J, Schubiger PA, Novak-Hofer I (2004) Hepatocyte growth factor-induced ectodomain shedding of cell adhesion molecule L1: role of the L1 cytoplasmic domain. J Biol Chem 279: 31149–31156. 15151998 10.1074/jbc.M403587200

[69] Owens RB (1974) Glandular epithelial cells from mice: a method for selective cultivation. J Natl Cancer Inst 52: 1375–1378. 4363715 10.1093/jnci/52.4.1375

[70] Owens RB, Smith HS, Hackett AJ (1974) Epithelial cell cultures from normal glandular tissue of mice. J Natl Cancer Inst 53: 261–269. 4366196 10.1093/jnci/53.1.261

[71] Yingling JM, Das P, Savage C, Zhang M, Padgett RW, et al (1996) Mammalian dwarfins are phosphorylated in response to transforming growth factor beta and are implicated in control of cell growth. Proc Natl Acad Sci U S A 93: 8940–8944. 8799132 10.1073/pnas.93.17.8940 PMC38573

[72] Hsu YH, Sarker KP, Pot I, Chan A, Netherton SJ, et al (2006) Sumoylated SnoN represses transcription in a promoter-specific manner. J Biol Chem 281: 33008–33018. 16966324 10.1074/jbc.M604380200

[73] Bondareva A, Downey CM, Ayres F, Liu W, Boyd SK, et al (2009) The lysyl oxidase inhibitor, beta-aminopropionitrile, diminishes the metastatic colonization potential of circulating breast cancer cells. PLoS One 4: e5620. 19440335 10.1371/journal.pone.0005620 PMC2680032

[74] Cailleau R, Young R, Olive M, Reeves WJ Jr (1974) Breast tumor cell lines from pleural effusions. J Natl Cancer Inst 53: 661–674. 4412247 10.1093/jnci/53.3.661 PMC7364228

[75] Eapen SA, Netherton SJ, Sarker KP, Deng L, Chan A, et al (2012) Identification of a Novel Function for the Chromatin Remodeling Protein ING2 in Muscle Differentiation. PLoS One 7: e40684. 22808232 10.1371/journal.pone.0040684 PMC3395697

[76] Scherr M, Morgan MA, Eder M (2003) Gene silencing mediated by small interfering RNAs in mammalian cells. Curr Med Chem 10: 245–256. 12570711 10.2174/0929867033368493

