# Supplementary figures and images for: Identification of a Novel Link between the Protein Kinase NDR1 and TGFβ Signaling in Epithelial Cells
Source: PLoS One. 2013 Jun 26;8(6):e67178. doi: 10.1371/journal.pone.0067178 (PMC3694053; doi:10.1371/journal.pone.0067178)

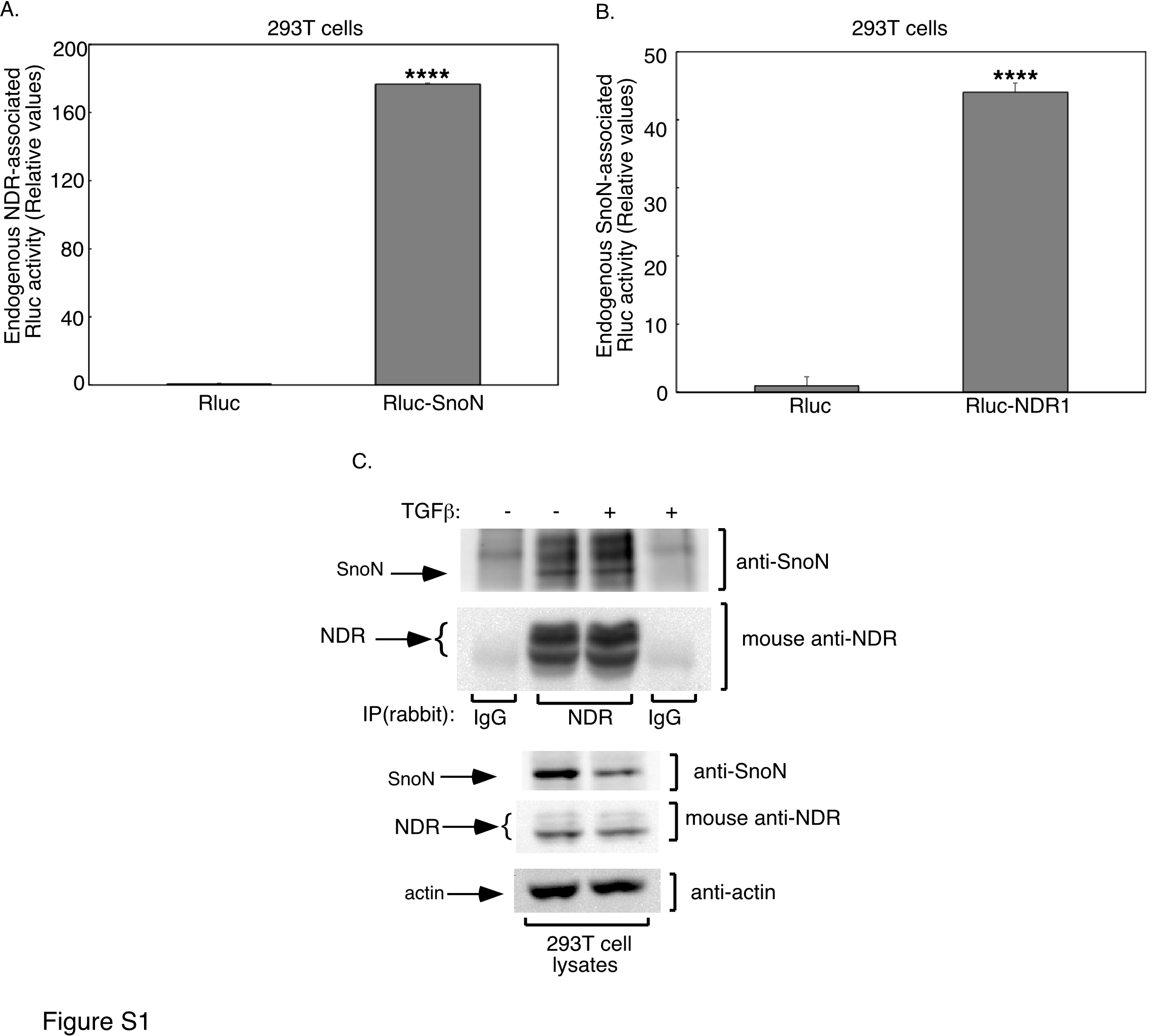

Supplement: Figure S1 — Related to Figure 1 . A. Lysates of 293T cells expressing Renilla luciferase (Rluc), alone, or as fusion with SnoN (Rluc-SnoN) were subjected to immunoprecipitation using the NDR1 antibody or IgG immunoglobulins, as a negative control, followed by analysis of immunoprecipitates by luciferase assays (90%) or immunoblotting (10%) with NDR1 antibody (data not shown). Cell lysates were also analyzed by luciferase assays and immunoblotting using NDR1 or actin antibody (data not shown). Endogenous NDR1-associated Rluc or Rluc-SnoN luciferase (IgG-subtracted) were normalized to Rluc or Rluc-SnoN, respectively, and endogenous NDR1 expression. The data are presented as the mean +SEM (n = 3) of NDR1-associated Rluc activity relative to Rluc activity associated with NDR1 in the case of the Rluc control. Rluc-SnoN associated robustly with endogenous NDR1. B. Lysates of 293T cells expressing Rluc or Rluc-NDR1 were subjected to immunoprecipitation using a SnoN antibody or IgG immunoglobulins, as a negative control, followed by analysis of the immunoprecipitates by luciferase assays (90%) or immunoblotting (10%) with SnoN antibody (data not shown). Cell lysates were also subjected to luciferase assays or immunoblotting with SnoN or actin antibody (data not shown). Endogenous SnoN-associated Rluc or Rluc-NDR1 activity was determined as in A. Data are presented as the mean+SEM (n = 4) of SnoN-associated Rluc activity relative to Rluc activity associated with SnoN in the case of the Rluc control. Rluc-NDR1 interacted strongly with endogenous SnoN. C. Lysates of untreated or TGFβ-treated 293T cells were subjected to immunoprecipitation using NDR1 antibody or IgG immunoglobulins, as a negative control, followed by immunoblotting with the SnoN or NDR1 antibody. Cell lysates were also subjected to immunoblotting with the SnoN, NDR1 or actin antibody with the latter serving as a loading control. **** in A and B indicates significant difference from the control (p<0.0001, t-test). (TIF) [file pone.0067178.s001.tif]

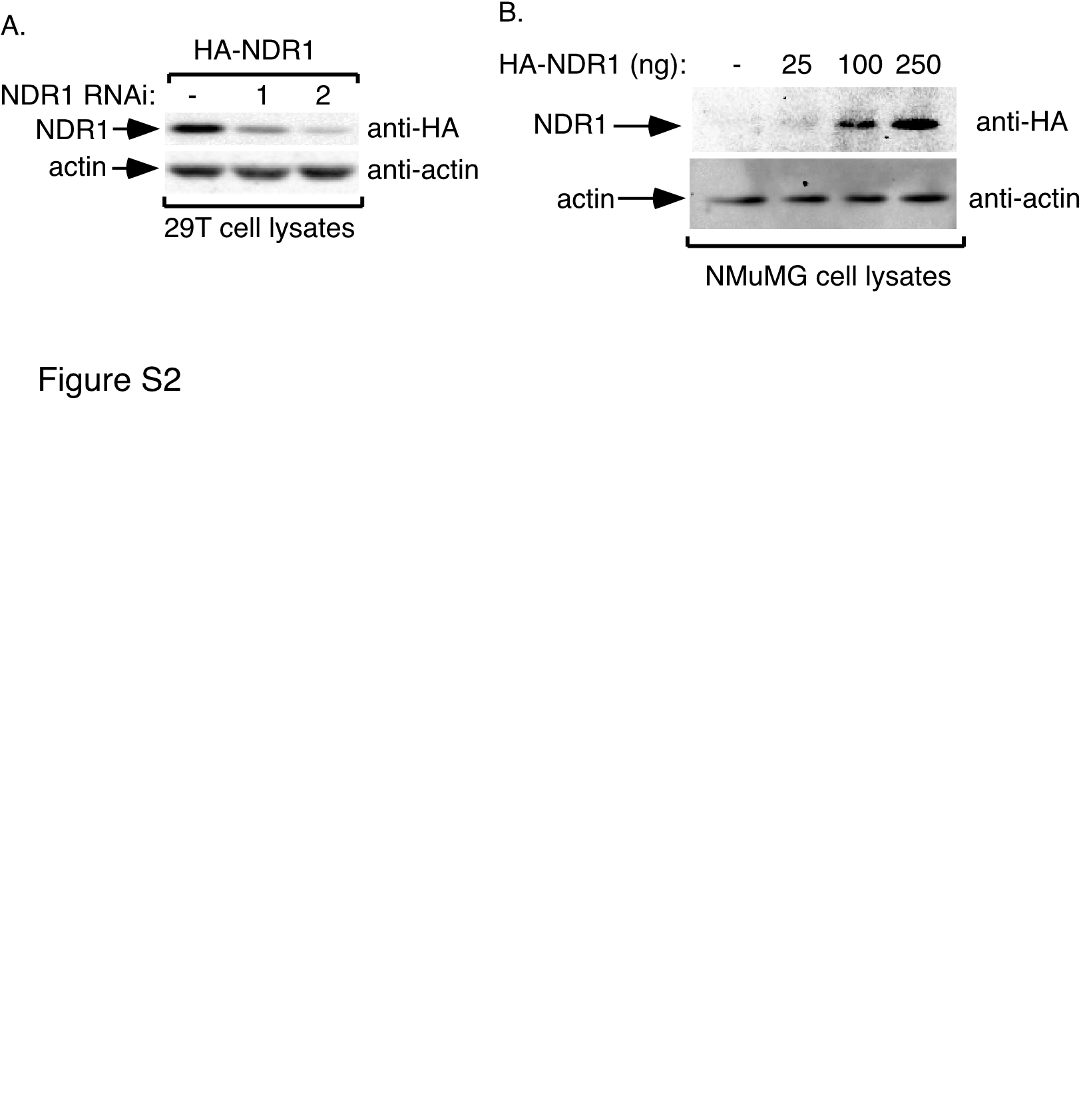

Supplement: Figure S2 — Related to Figure 2 . A. Lysates of 293T cells expressing HA-NDR1 in the presence of the control RNAi vector, or NDR1 RNAi NDR1i-1 or NDR1i-2 plasmid were subjected to immunoblotting using the HA or actin antibody, with the latter to serve as a loading control. NDR1i-1 or NDR1i-2 induced 80 to 90 percent knockdown of NDR1. B. Lysates of NMuMG cells transfected with increasing concentrations of a plasmid expressing HA-NDR1 together with the TGFβ-responsive 3TP-luciferase reporter and a transfection efficiency vector as described in Figure 2C, were subjected to immunoblotting using the HA or actin antibody. Images in A and B are representative blots from experiments that were repeated at least two independent times. (TIF) [file pone.0067178.s002.tif]

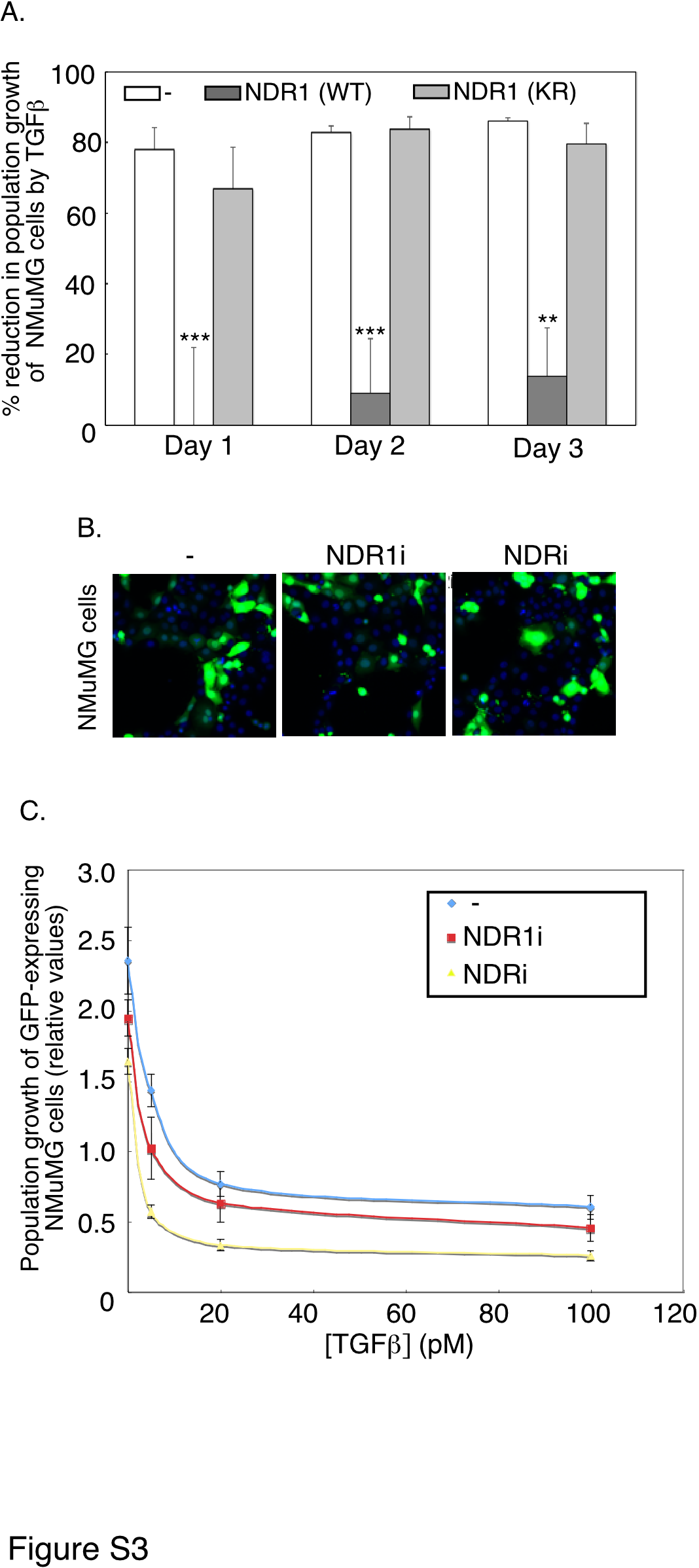

Supplement: Figure S3 — Related to Figure 4 . A. Population growth of NMuMG cells expressing wild type (WT) or kinase-inactive (KR) NDR1, or control vector (−) after culturing for one, two, or three days in the absence or presence of 100 pM TGFβ was determined by subjecting DNA dye (Hoechst)-labeled NMuMG cells to fluorescence microscopy and data analysis using the Cellomics KSR platform and Target Activation algorithm. Percent decrease in population growth of NMuMG cells by TGFβ was quantified as described in Figure 4B. Data are presented as the mean+SEM of percent reduction of population growth of NMuMG cells by TGFβ from three (day 1 and day 3) or five (day 2) independent experiments. ** or *** indicates significant difference from the respective control within each day at p<0.01, or P<0.001, respectively (ANOVA). B. Representative fluorescence images of NMuMG cells one day post transfection with control RNAi, NDR1i or NDRi plasmids as described Figure 4E, where the DNA dye Hoechst (blue) and GFP (green)-induced signals indicate total NMuMG cells and transfected NMuMG cells, respectively. Analysis of the GFP-labeled cells as compared to total cells using the target activation algorithm indicated approximately 50 percent transfection efficiency for all three sets of transfections. The width of each micrograph corresponds to 330 µm. C. For each experiment including the one shown in Figure 4E, triplicate average of GFP-positive cells at each TGFβ concentration was determined. Data are presented as the mean±SEM of relative GFP-positive cell numbers from six (control and NDRi) or five (NDR1i) independent experiments. (TIF) [file pone.0067178.s003.tif]

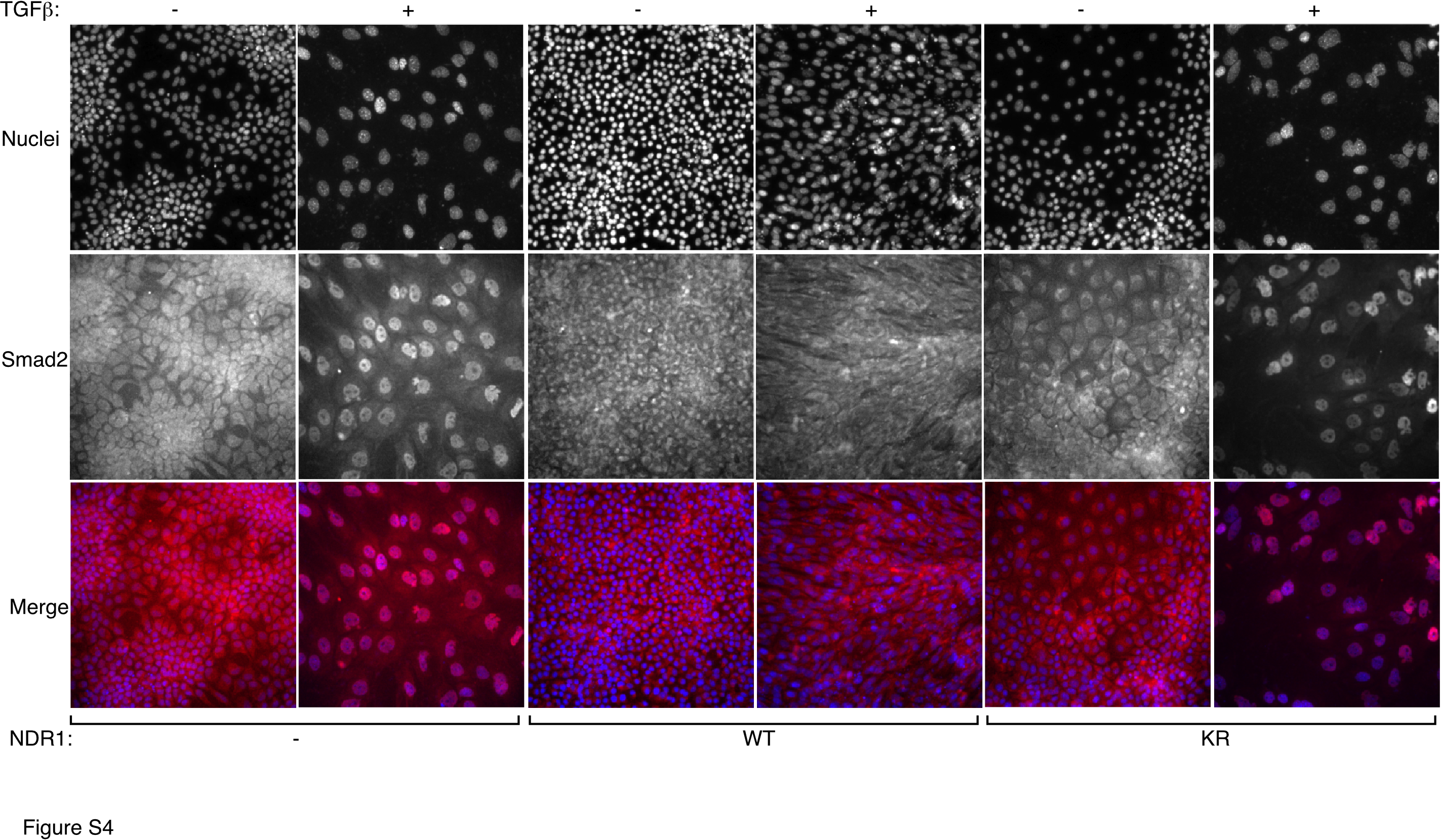

Supplement: Figure S4 — Related to Figure 5 . Representative images of untreated or TGFβ-treated NMuMG cells expressing wild type or kinase-inactive NDR1 or vector control that were subjected to indirect immunofluorescence using the Smad2 antibody and a Cy3-secondary antibody (red) and labeling with the DNA Hoechst dye (blue), and scanned by fluorescence microscopy. The width of each micrograph corresponds to 330 µm. (TIF) [file pone.0067178.s004.tif]

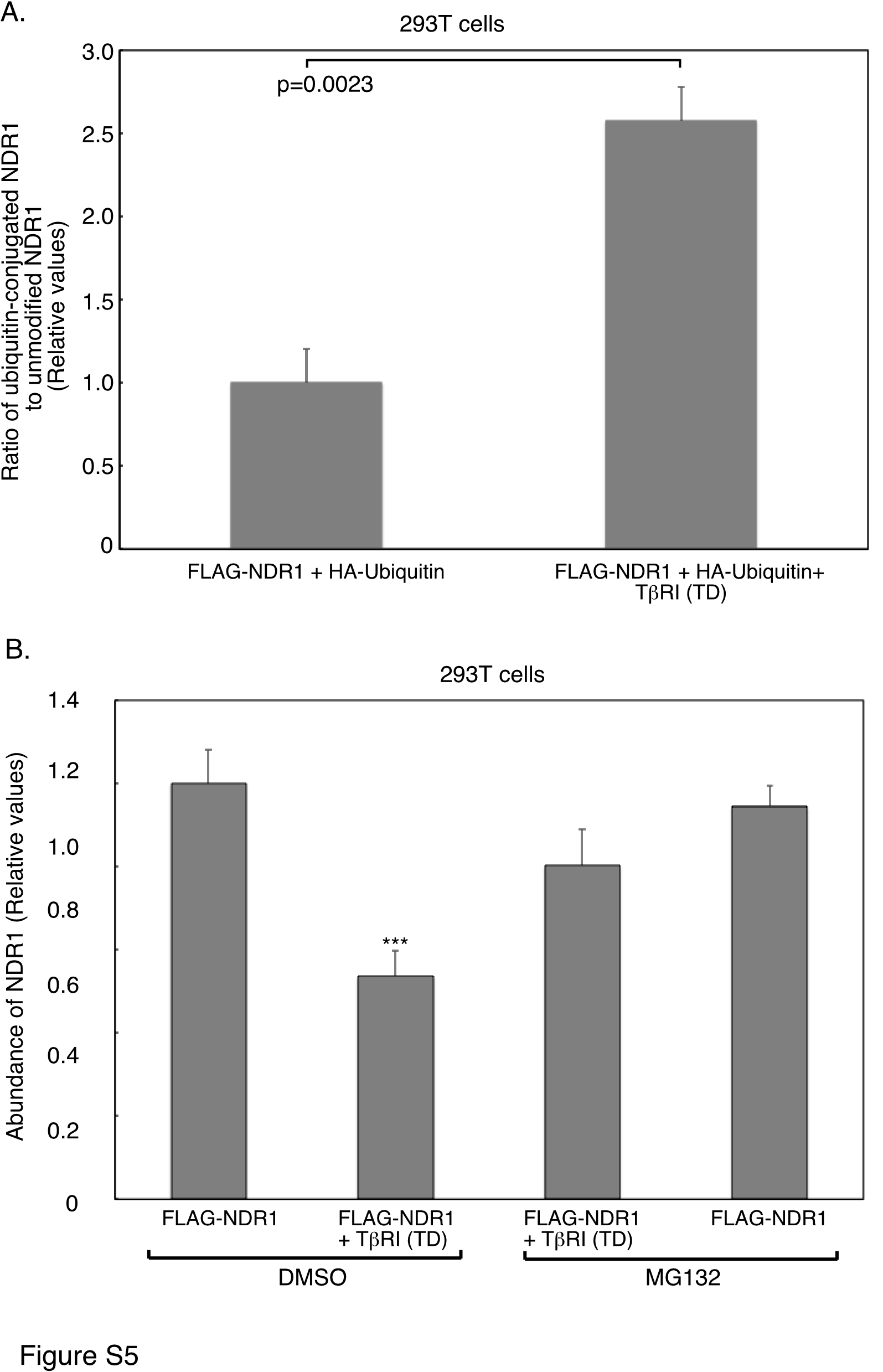

Supplement: Figure S5 — Related to Figure 6 . A. Lysates of 293T cells coexpressing FLAG-NDR1 and HA-ubiquitin alone or together with the constitutively active TGFβ type I receptor, harboring a mutation in Threonine 204 to aspartate, were subjected to immunoprecipitation using the FLAG antibody followed by immunoblotting with the HA or NDR1 antibody as described in Figure 6H. Ubiquitin-conjugated NDR1 protein species as indicated in and including the protein species in Figure 6H immunoblots were quantified and normalized to NDR1 levels in the immunoprecipitates. Data are presented as the mean+SEM (n = 3) of ubiquitin-conjugated NDR1 species relative to the ubiquitinated NDR1 in cells coexpressing NDR1 and ubiquitin. Significant difference between the two groups was determined using unpaired, two-tailed t-test. B. Lysates of untreated or MG132-treated 293T cells expressing FLAG-NDR1 alone or together with constitutively active receptor were subjected to FLAG and actin immunoblotting as described in Figure 6I. NDR1 protein species as indicated and including the protein species in Figure 6I were quantified and normalized to respective actin. Data are presented as the mean+SEM (n = 6) of NDR1 relative to NDR1 in cells expressing NDR1 alone and left in the absence of MG132. *** indicates significant difference from the control (p<0.001, ANOVA). (TIF) [file pone.0067178.s005.tif]

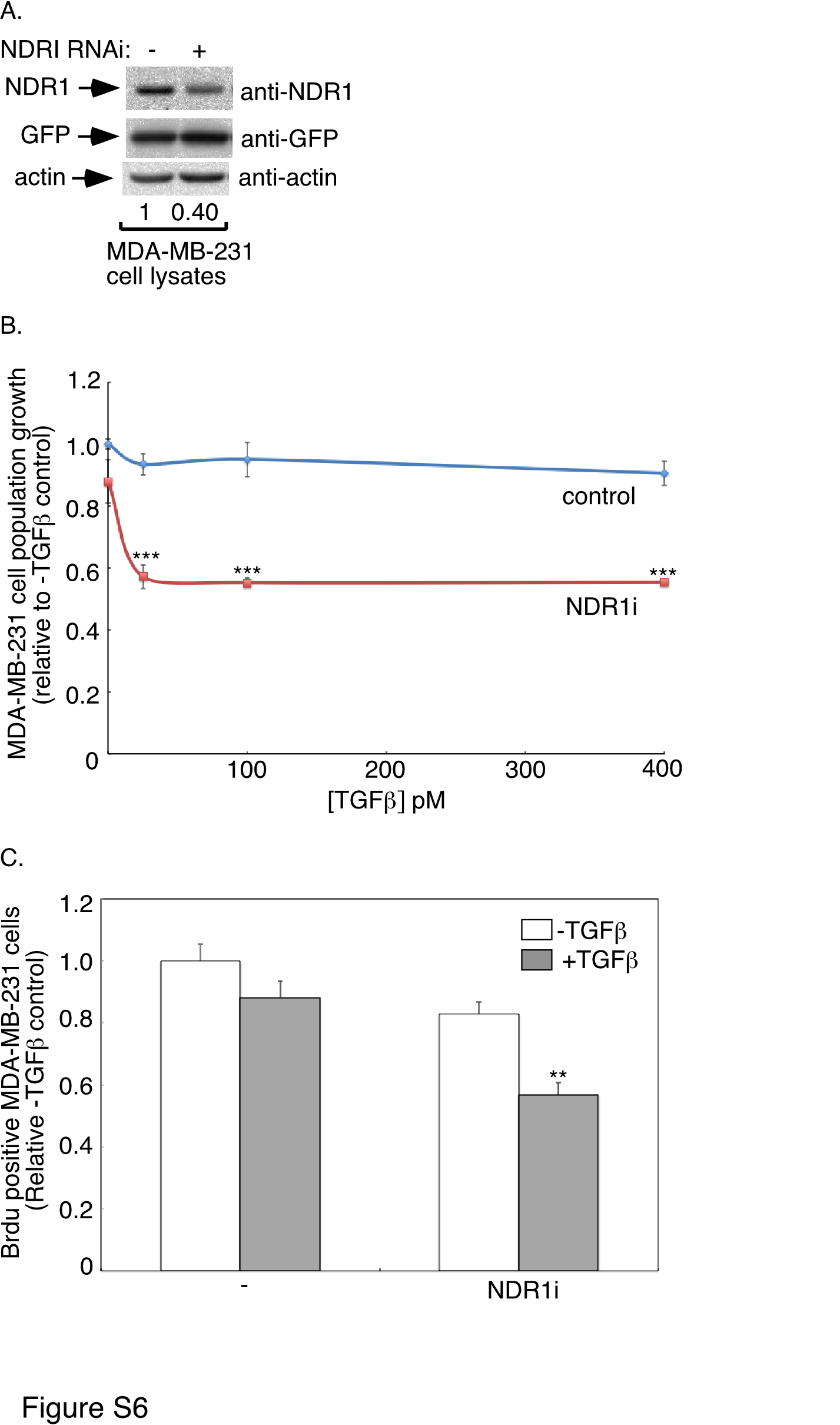

Supplement: Figure S6 — NDR1 knockdown restores the ability of TGFβ to inhibit cell proliferation in the breast MDA-MB-231 carcinoma cells. A. Lysates of MDA-MB-231 transfected with a control or NDR1 RNAi plasmids, were subjected to immunoblotting with an NDR1 or actin antibody. Values shown below lanes 1 and 2 represent actin-normalized NDR1 level expressed relative to the actin-normalized NDR1 level in the RNAi control vector transfected cells. B. GFP-expressing and DNA-Hoechst-labeled MDA-MB-231 cells transfected as in A and incubated one day post transfection with 0, 25, 100, or 400 pM TGFβ for 72 h were imaged and quantified by fluorescence microscopy and the target activation bio-application, respectively, using the Cellomics KSR as in Figure 4 and Figure S3. Untreated or TGFβ-treated cells were seeded in triplicates or quadruplicates in a 96-well plate, and population growth of GFP-positive cells were averaged. Data are presented as the mean+SEM of average population growth of GFP-positive MDA-MB-231 cells from five independent experiments expressed relative to the untreated control. C. MDA-MB-231 cells transfected as in A and left untreated or treated with 400 pM TGFβ were incubated for the last hour with bromodeoxyuridine, and subjected to immunocytochemistry using a BrdU antibody, fluorescence microscopy and analysis as described in Figure 4. Target activation bioapplication was used to quantify ratio of GFP-expressing BrdU labeled cells, and averages of replicates quantified as in B. Data are presented as the mean+SEM of GFP-expressing BrdU-positive cells from 5 independent experiments. ** or *** indicates significant difference from the control at p<0.01, or p<0.001, respectively (ANOVA). (TIF) [file pone.0067178.s006.tif]
